# Molecular docking and experimental evaluation of natural alkaloids from Chilean flora (*Cryptocarya alba*, *Peumus boldus*, and *Laurelia sempervirens*) for tyrosinase inhibition and depigmenting potential

**DOI:** 10.3389/fphar.2026.1795496

**Published:** 2026-04-14

**Authors:** Sebastián Castro-Saavedra, Gonzalo Fuentes-Barros, Marco Mellado, Javier Echeverría

**Affiliations:** 1 Departamento de Ciencias del Ambiente, Facultad de Química y Biología, Universidad de Santiago de Chile, Santiago, Chile; 2 SAPHYCHEM, Santiago, Chile; 3 Programa de Doctorado en Políticas Públicas, Universidad Mayor, Santiago, Chile; 4 Dirección de Investigación, Universidad Bernardo O’Higgins, Santiago, Chile; 5 Laboratorio de Espectroscopia y Química Aplicada, Grupo de Investigación en Ciencias Biomédicas Aplicadas, Universidad Central de Chile, Santiago, Chile

**Keywords:** aporphine alkaloids, benzylisoquinoline alkaloids, *Cryptocarya alba*, *Laurelia sempervirens*, *Peumus boldus*, skin depigmentation, tyrosinase activity

## Abstract

**Background:**

Hyperpigmentation disorders stem from tyrosinase-catalyzed melanin overproduction, worsened by ultraviolet oxidative stress. The native Chilean species *Cryptocarya alba*, *Laurelia sempervirens*, and *Peumus boldus* are sources of aporphine and benzylisoquinoline alkaloids, known for their antioxidant activity. Boldine derivatives, notably diacetylboldine (DAB, Lumiskin™), demonstrate commercial depigmenting efficacy, yet structure-activity relationship data are limited.​

**Purpose:**

Systematically evaluate pure alkaloids and boldine derivatives for tyrosinase inhibition *via* mushroom enzyme assays, molecular docking, human melanocyte (HEMn-DP), and 3D epidermal (MelanoDerm™) models to elucidate depigmenting mechanisms.

**Material and Methods:**

Boldine (BOL), *N*-methyllaurotetanine (NMLT), laurolitsine (LTS), laurotetanine (LTT), and reticuline (RET) alkaloids were isolated from authenticated plant material *via* acid–base partitioning/silica gel chromatography; structures were confirmed by ^1^H-NMR/UHPLC–MS/MS (98% HPLC purity). Coclaurine (CC), *N*-methylcoclaurine (NMCC), and BOL derivatives: 3-bromoboldine (3BrBOL) and DAB were synthesized. Isocorydine (ISO) was obtained commercially. Mushroom tyrosinase (EC 1.14.18.1) inhibition measured spectrophotometrically (475 nm, L-DOPA; IC_50_ interpolation). Docking performed on *A. bisporus* tyrosinase (PDB:2Y9X) with AutoDock Vina (30 × 30 × 30 Å grid). HEMn-DP cells treated (40/250 ppm, 24 h) for MTS viability and alkali-solubilized melanin (450 nm). MelanoDerm™ tissues were dosed topically (0.1% w/v) for 14 days, followed by the Solvable™ melanin assay (490 nm). Data analyzed in GraphPad Prism 8 (Dunnett’s test, *P* < 0.05).

**Results and Discussion:**

LTS (IC_50_ = 0.96 mM), an aporphine alkaloid, followed by CC (1.29 mM), a benzylisoquinoline alkaloid, were the most potent mushroom tyrosinase inhibitors, outperforming BOL 6.8- and 5.1-fold (6.56 mM), respectively, yet trailing reference standards such as kojic acid (0.014 mM). Boldine derivatives, including DAB (2.22 mM) and 3BrBOL (1.96 mM), exhibited superior potency relative to the parent compound. Docking revealed LTS’s highest affinity (−7.3 kcal/mol; H-bonds: His259 3.4 Å, Asn260 3.2 Å) *versus* BOL (−5.3 kcal/mol; solely van der Waals), explained by NH-mediated His263 interaction absent in *N*-methylated boldine. In addition, hemisynthetic alkaloids (3BrBOL and DAB) show interactions and affinity energies similar to those of BOL. In HEMn-DP (40 ppm, 24 h), BOL/3BrBOL achieved 100% tyrosinase inhibition (viability ∼50%), exceeding DAB (62%). MelanoDerm™ (0.1% w/v, 14 days): results confirmed the efficacy of 3BrBOL (−33% melanin, *P* < 0.05; ∼arbutin), BOL (−10%), and DAB/LTS inactivity, highlighting the superiority of C-3 halogenation.

**Conclusion:**

LTS is the premier natural inhibitor; optimization of 3BrBOL validates BOL derivatization for ARB equivalent depigmentation in 3D models. These findings support the development of hydroquinone-free cosmeceuticals from Chilean flora alkaloids and advocate the use of nanoemulsions to enhance delivery and reduce cytotoxicity.

## 1 Introduction

Skin aging is a multifactorial, complex process involving multiple biological mechanisms and theories, as well as determinants such as gender, ethnicity, and genetic predisposition. Among extrinsic factors, excessive exposure to ultraviolet (UV) radiation is the most significant cause of photoaging, inducing oxidative stress, DNA damage, and dermal collagen degradation. This process accelerates the visible signs of aging, promoting the appearance of localized hyperpigmentation and age spots (Fisher et al., 2002; Zouboulis, 2007; Poljšak and Dahmane, 2012; Alves et al., 2013; Tobin, 2017). Hyperpigmentation results from excessive melanin accumulation in the basal layer of the epidermis. Melanin is synthesized in melanosomes, specific organelles produced by melanocytes located in the basal layer. Subsequently, melanosomes transport melanin to keratinocytes, where the pigment is distributed to the stratum corneum. This mechanism has been documented in various scientific sources, which explain that hyperpigmentation, such as post-inflammatory hyperpigmentation, is characterized precisely by this increase in pigment in the epidermal basal layer (Khmaladze et al., 2020a).

*Cryptocarya alba* (Mol.) Looser [Lauraceae], *Laurelia sempervirens* (Ruiz et Pav.) Tul. [Atherospermataceae], and *P. boldus* Mol. [Monimiaceae] are woody trees native to the sclerophyllous and temperate forests of central and southern Chile (Gajardo, 1995; Luebert and Pliscoff, 2006), which share a rich profile of secondary metabolites, in particular aporphine and benzyl-tetrahydroisoquinoline alkaloids, as well as terpenes and polyphenols present in leaves, bark, and fruits (Fuentes-Barros et al., 2018; 2025; Giordano et al., 2019; Touma et al., 2020). Among them, *P. boldus* stands out as one of the best-studied medicinal species in the country and is recognized at the regulatory level, with leaves included in pharmacopoeias and official monographs due to their choleretic, hepatoprotective, and antioxidant effects (Speisky and Cassels, 1994; Cassels et al., 2019). Meanwhile, *C. alba* and *L. sempervirens*, despite their prolonged use in traditional medicine, still require further pharmacological validation and translational studies targeting specific areas such as tyrosinase and melanogenesis, where plant-derived products can contribute (Schmeda-Hirschmann et al., 1994; Carimonei, 2024; Fuentes-Barros et al., 2025).

In particular, for *P. boldus*, clinical studies remain limited (Gotteland et al., 1995), with preclinical trials predominating and supporting its multifunctional profile (Cassels et al., 2019; 2021). Its cytoprotective properties against various toxins are particularly noteworthy (Cassels et al., 2019; 2021). Furthermore, boldo possesses diuretic, anti-inflammatory, antioxidant, analgesic, and antispasmodic effects, which support its use in gastrointestinal, hepatic, and genitourinary conditions (Speisky and Cassels, 1994; Fernández et al., 2009; Figueiredo et al., 2016; Cassels et al., 2019; 2021; Abouelela et al., 2023).

Boldo biomass contains high levels of secondary metabolites, with concentrations varying significantly with factors such as sex, harvest time, organ type, and plant age. It is particularly noteworthy as a source of alkaloids such as boldine, laurolitsine, *N*-methyllaurotetanine, isocorydine, and coclaurine (Fuentes-Barros et al., 2018; 2023). Boldine is the best-known alkaloid of this species, as it is the main compound extracted from its bark and is representative of the type of alkaloids responsible for many of the medicinal properties of the leaves (Cassels et al., 2021).

Currently, there are few cosmetic products on the market made from *P. boldus* biomass, such as BASF’s Betapur™ (Leaf extract), which regulates the skin microbiota by activating β-defensins and conferring purifying and anti-acne properties.

Vegetables, fruits, and medicinal plants rich in phenolic compounds and antioxidant vitamins can help mitigate the effects of skin aging, oxidative stress, and photodamage, and restore skin barrier function (Akalın and Selamoglu, 2019; Wan et al., 2023; Tomas et al., 2025). Various natural ingredients exert beneficial effects on the skin through anti-inflammatory and pro-healing mechanisms associated with repair processes and oxidative stress that frequently coexist with hyperpigmentation, such as royal jelly, melittin (Ravichandran and Selamoglu, 2023), and alkaloids like berberine (A Okoye et al., 2025). Therefore, their medical application can offer many benefits for the treatment of skin conditions (Ren et al., 2025). Given this potential, there is growing interest in multifunctional natural active ingredients for topical use, including anti-inflammatory, antioxidant, and depigmenting agents. In this sense, natural products (Peralta et al., 2025), such as alkaloids, constitute a family of bioactive ingredients targeting tyrosinase. An *in silico* study analyzed the main compounds naturally present in three botanical species commonly used in traditional Chinese medicine for skin bleaching: *Ampelopsis japonica* (Thunb.) Makino [Vitaceae], *Lindera aggregata* (Sims) Kosterm. [Lauraceae], and *Ginkgo biloba* L. [Ginkgoaceae]. In a theoretical human tyrosinase homology docking model (Surflex-Dock), nine components—five of them alkaloids—from these herbs showed higher binding energies than the reference bleaching agents, arbutin and kojic acid (Fong and Tong, 2012).

Aporphine alkaloids such as nuciferine, the main phytochemical of *Nelumbo nucifera* Gaertn [Nelumbonaceae], exhibit a remarkable antioxidant capacity by eliminating oxygen and hydroxyl free radicals, as well as anti-inflammatory and anticancer effects (Veerichetty et al., 2023). Methanolic extracts of this species inhibit theophylline-induced melanogenesis in murine melanoma B16 4A5 cells, with nuciferine and *N*-methylassimilobin showing potencies of 3–30 μM by reducing the expression of tyrosinase mRNA, TRP-1, and TRP-2 (Nakamura et al., 2013). Berberine, an isoquinoline alkaloid with antibacterial, anti-inflammatory, and lipid-lowering properties, attenuates, in a dose-dependent manner, the expression of MITF and tyrosinase, and consequently, of TRP-1, in α-MSH-stimulated B16F1/F10 cells, inhibiting the PI3K/AKT, ERK, and GSK3β pathways, as well as pigmentation in zebrafish (*Danio rerio*) models (Song et al., 2015; Lee et al., 2018). This pharmacological background underscores the depigmenting potential of natural alkaloids.

In this context, other aporphines such as 3-aminomethylglaucine (Chochkova et al., 2020) and boldine have been proposed as potential depigmenting agents, in the case of boldine stands out as a mixed inhibitor of tyrosinase, capable of interacting with both the free enzyme and its complex with L-DOPA, positioning itself as a potential depigmenting agent (Si et al., 2013). Likewise, the Lumiskin™ and Lumisphere™ products developed by Sederma (Croda Group) are the most recognized boldo-derived products: both contain diacetyl boldine (DAB), while Lumisphere™ combines DAB with reflective microspheres that provide immediate luminosity to the skin. This development stems from research by Mas-Chamberlin and colleagues, who proposed that noraporphine derivatives interfere with calcium influx and adrenergic signaling, thereby inhibiting tyrosinase function and reducing melanin synthesis for dermocosmetic applications. Among these compounds, DAB demonstrated *in vitro* inhibition of melanogenesis and was developed for use in topical depigmenting formulations. Specifically, studies in murine B16 melanocytes (IC_50_ ∼20 ppm) and human melanocytes (IC_50_ ∼40 ppm) reported significant reductions in melanogenesis at low concentrations, with an IC_50_ for kojic acid of approximately 1,000 ppm; this reduction was reversible (Mas-Chamberlin et al., 2004). Additionally, DAB inhibited melanogenesis at 10 and 40 ppm in SkinEthic® three-dimensional epidermal models, as confirmed by histological sections on day 7 of incubation.

These results were confirmed in another cosmetic study (Morganti, 2015). Consistent with preclinical evidence, clinical evaluations in Asian and Caucasian populations have observed marked inhibition of melanogenesis following topical application of DAB formulations, supporting their efficacy in cosmetic hyperpigmentation scenarios (Mas-Chamberlin et al., 2004). Other researchers found that the combined treatment of DAB and the biomimetic oligopeptide TGF-β1-68 is more effective and safer for facial melasma than hydroquinone, showing significant improvement and good skin tolerance in clinical studies (Pratchyapurit, 2016). Furthermore, a cosmetic formulation containing plant extracts and DAB was evaluated against a 3% vitamin C derivative in adult Japanese volunteers with melasma and/or age spots. After 8 weeks of bilateral facial application, both formulations significantly improved pigmented spots and skin radiance, with comparable efficacy as assessed by clinical assessments and questionnaires (Tadokoro et al., 2010). In a multipurpose formulation (antipollution, brightening, and antiaging) with active ingredients tested separately, active ingredient B, consisting of caprylic/capric triglyceride and DAB, showed an antimelanogenic effect in *in vitro* primary melanocyte monocultures and 3D spheroid models. It was most effective at suppressing MITF gene expression in melanocytes (a key regulator of melanogenesis) (Khmaladze et al., 2020b). DAB is also combined with Sepiwhite® and salicylic, pyruvic, and all-*trans*-retinoic acids to treat hyperpigmentation and irregular pigmentation (Wong, 2021). Recently, a microemulsion with DAB was developed and optimized for topical administration, and its permeation and cytotoxicity in B16BL6 cells were evaluated. Two formulations with a particle size of 50 nm showed greater skin retention and higher cytotoxicity, as assessed by the MTT assay, with an inhibitory concentration of 50% (IC_50_) up to 50 times that of the control (DAB in oil) (Al Saqr et al., 2023).

In this context, the present work focuses on obtaining and evaluating individual alkaloids from Chilean species—boldine (BOL), laurolitsine (LTS), and *N*-methyllaurotetanine (NMLT) (*P. boldus*), reticuline (RET) (*C. alba*), and laurotetanine (LTT) (*L. sempervirens*)—as well as synthesizing coclaurine (CC) and *N*-methylcoclaurine (NMCC), and boldine synthetic analogs: DAB and 3-bromoboldine (3BrBOL). First, the main alkaloids from native Chilean plants were identified; second, their enzymatic inhibition on mushroom tyrosinase was evaluated; third, a molecular docking study was conducted on *Agaricus bisporus* tyrosinase (PDB 2Y9X) to determine their potential as melanogenesis inhibitors and elucidate structural interactions at the active site; and fourth, cytotoxicity and melanin inhibition assays were carried out in human HEMn-DP melanocytes (neonatal, darkly pigmented), along with melanin content evaluation in 3D MelanoDerm tissue models using BOL, DAB, and 3BrBOL.

## 2 Materials and methods

### 2.1 Materials

Mushroom tyrosinase (EC 1.14.18.1), melanin, silica gel 60 Å (40–63 µm), arbutin (ARB), kojic acid (KA), gallic acid (GA), PBS, NaOH, and L-DOPA were purchased from Sigma-Aldrich (St. Louis, MO, United States). Monosodium phosphate, disodium phosphate, anhydrous sodium sulfate, methanol (MeOH), dichloromethane (DCM), hydrochloride chloride (HCl), ethyl acetate (EtOAc), ammonium hydroxide (NH_4_OH), trichloromethane (CHCl_3_), *N*-bromosuccinimide, trifluoroacetic acid (TFA), pyridine, acetic anhydride and dimethyl sulfoxide (analytical grade), Dragendorff’s reagent spray solution and thin-layer chromatography (TLC) were purchased from Merck (Darmstadt, Germany). Isocorydine HCl was purchased from PhytoLab GmbH & Co. (Vestenbergsgreuth, Germany) (Purity >98% by HPLC). All solvents used were of analytical grade. Water was of ultrapure grade.

### 2.2 Plant material and extraction for alkaloid isolation

Leaves, woods, and bark of *P. boldus* were collected near María Pinto (33°26′S, 71°18′W; 230–250 m.a.s.l.), Santiago Metropolitan Region, Chile. Dried materials were extracted three times (4 h each) with MeOH (5 L/kg) at 50 °C. The combined liquors were filtered and concentrated to yield dark gummy residues, which were suspended in DCM and partitioned with 1 M aqueous HCl. The aqueous phase was washed sequentially with hexane, EtOAc, and DCM, then basified to pH 9–10 with 25% aq. NH_4_OH, and extracted three times with DCM. The combined organic extracts were dried (Na_2_SO_4_), filtered, and evaporated. Total alkaloid extracts were fractionated by silica gel 60 Å (40–63 μm; 30 × 3, 30 × 5, and 30 × 7 cm columns) using an EtOAc:MeOH gradient (100:0 → 0:100), collecting ca. 100 fractions of 20 mL each. Fractions were monitored by TLC (EtOAc: MeOH 4:1 or DCM:MeOH 5:3; UV 254/365 nm; Dragendorff’s reagent). From 100 g of *P. boldus* bark methanolic extract, acid-base partitioning followed by recrystallization from CHCl_3_ yielded boldine (BOL) (0.4% yield). Further purification of the wood extract by silica gel column chromatography (DCM:MeOH 5:3) afforded laurolitsine (LTS) (0.25% yield).

**FIGURE 1:**
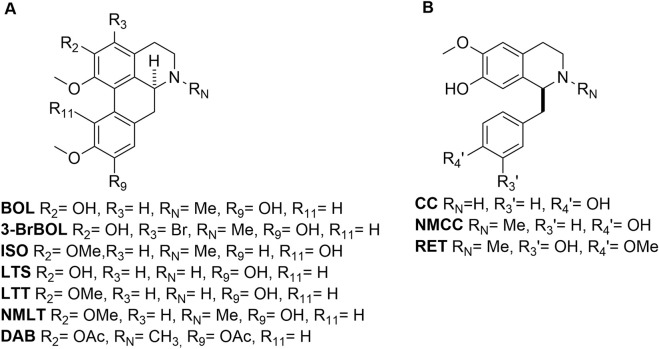
Chemical structures of the compounds studied. **(A)** Aporphines: boldine (BOL), 3-bromoboldine (3BrBOL), isocorydine (ISO), laurolitsine (LTS), laurotetanine (LTT), *N*-methyllaurotetanine (NMLT), diacetylboldine (DAB). **(B)** Benzylisoquinolines: (*R,S*)-coclaurine (CC), (*R,S*)-*N*-methylcoclaurine (NMCC), and (*R,S*)-reticuline (RET).

From 2.2 kg of *P. boldus* leaves, pressurized hot water extraction (50 g batches in 1 L) gave an aqueous extract that was basified (NH_4_OH, pH 9), extracted with CHCl_3_, and concentrated to an amorphous solid (19.319 g). Acid-base partitioning (1 M HCl, then NH_4_OH to pH 9, CHCl_3_ extraction) yielded a powder that was fractionated by silica gel column chromatography (EtOAc:MeOH 4:1), affording five fractions. Fraction 2 (Rf 0.7; 1.495 g) was rechromatographed (DCM:MeOH 5:3), and subfraction 2 (Rf 0.6; 867 mg) was further purified (DCM:MeOH 9:1) to yield *N*-methyllaurotetanine (NMLT) (110 mg). Reticuline (RET) was isolated as described previously from the bark of *C. alba* (Castro-Saavedra et al., 2016), and laurotetanine (LTT) was isolated from the bark of *L. sempervirens* (Fuentes-Barros et al., 2018). Structures of all compounds (Purity >98% by HPLC) (Figure 1) were confirmed by ^1^H NMR spectroscopy and UHPLC-MS/MS analysis (molecular ion and fragmentation patterns, Supplementary Table S1; Supplementary Figures S1–S8), with spectral data matching those reported in the literature (Tomita and Kugo, 1956; Guinaudeau et al., 1975; Hara et al., 1995; Sobarzo-Sánchez et al., 2000; Castro-Saavedra et al., 2016; Al-ghazzawi, 2019).

### 2.3 Synthesis of derivatives and other reference standards

3-Bromoboldine (3BrBOL) was synthesized from BOL and *N*-bromosuccinimide in TFA at 20 °C. After 1 h of stirring, cold water was added, the pH was adjusted to 8–9 (NH_4_OH), and the product was extracted with CHCl_3_. Optimized reagent stoichiometry afforded the 3-monobrominated derivative (Sobarzo-Sánchez et al., 2000). 2,9-*O,O*-diacetylboldine (DAB) was prepared from BOL, pyridine, and acetic anhydride using a previously described methodology (Thomet et al., 2010), affording the product in moderate yield. (*R*,*S*)-coclaurine (CC) and (*R*,*S*)-*N*-methylcoclaurine (NMCC) were synthesized by the Bischler-Napieralski route (Teitel and Brossi, 1968). The synthesized compounds were confirmed by UHPLC-MS/MS analysis (molecular ion and fragmentation patterns, Supplementary Table S1) and ^1^H NMR spectroscopy (Supplementary Tables S2 and S3).

### 2.4 Liquid chromatography and mass spectrometry

Samples were analyzed by an ultra-high-pressure liquid chromatograph (UHPLC) model (Eksigent model EkspertUltraLC 100-XL) coupled to a triple quadrupole mass spectrometer in the electrospray mode (ESI) (ABSciex Triple Quad 4500). Column: Phenomenex Synergi™ Fusion-RP 80 Å (50 mm × 2.0 mm, 4 µm); injection: 10 μL. The mobile phases: A = 0.1% v/v formic acid 0.1% v/v and B = acetonitrile; flow: 0.3 mL/min. Gradient program: 0 min 3% B, 3 min 3% B, 13 min 15% B, 16 min 20% B, 17 min 3%, 18 min 3% B. MS parameters: GS1 nitrogen 40 psi, GS2 nitrogen 50 psi, CURT nitrogen 25 psi, IS 3500 V, TEMP 650 °C. Data Analyst 1.6.2 and the data processed by Multiquant 3.0. (Fuentes-Barros et al., 2018; 2023). Fragmentation patterns were reviewed in available databases, and when this information was not found, the scientific literature was consulted; the presence of at least three matching fragmentation patterns was considered an acceptance criterion. The databases used were hmdb.ca, MassBank.eu, mzCloud.org, and mona.fiehnlab.ucdavis.edu.

### 2.5 ^1^H NMR spectroscopy conditions

NMR spectra were recorded at 300 K in CDCl_3_ at 400 (^1^H) on a Bruker Avance III HD 400 MHz spectrometer (Bruker AXS GmbH, Karlsruhe, Germany).

### 2.6 Tyrosinase inhibition

All measurements were performed using a Spectroquant® Pharo 300 spectrophotometer Merck (Darmstadt, Germany) at 25 °C. A stock solution of mushroom tyrosinase (5771 units per mL, 8.06 µM) was stored at −20 °C prior to use. All inhibitor solutions were prepared from the free base by mixing 1.8 mL of 0.1 M phosphate buffer, 0.6 mL of milliQ water, 0.1 mL of the sample dissolved in 1 mL of DMSO (HPLC grade, <3.3% v/v), and 0.1 mL of tyrosinase stock solution. This mixture was pre-incubated for 5 min at 25 °C before adding an aliquot of a light-protected L-DOPA solution (6.3–22.1 mM in the spectrophotometer cuvette). To validate the method, ARB, KA, and GA were used as positive controls. Tyrosinase activity was evaluated by monitoring absorbance at 475 nm over approximately 10 min. All IC_50_ values were obtained by interpolation from the plot of inhibitor concentration *versus* enzymatic inhibition (Kubo and Kinst-Hori, 1998).

### 2.7 Molecular docking

The molecular docking analysis was conducted following the previously described method with minor adjustments (Araya et al., 2025). The three-dimensional structure of *Agaricus bisporus* tyrosinase was retrieved from the Protein Data Bank (PDB ID: 2Y9X) (Ismaya et al., 2011). The crystallized ligand (0TR, tropolone) was removed using ArgusLab 4.0.1 (Mark Thompson and Planaria Software LLC, Seattle, WA, United States, 1997–2004). The resulting apoenzyme structure was subsequently imported into AutoDock Tools 1.5.6 (MGLTools), where polar hydrogen atoms were added, Gasteiger–Hückel charges were assigned, and the file was saved in .pdbqt format.

Ligands were constructed with ChemDraw 3D 15.1.0.144 (PerkinElmer Informatics, Inc., Waltham, WA, United States, 1998–2016), ensuring the correct assignment of all stereogenic centers. The structures were exported in .mol2 format and further processed in AutoDock Tools 1.5.6 (Molecular Graphics Laboratory, The Scripps Research Institute, La Jolla, CA, United States, 1999–2011).

The docking grid was centered on the native ligand coordinates (x = −4.64, y = −25.43, z = −35.03) and had a box size of 30 Å × 30 Å × 30 Å along the x, y, and z-axes, respectively. These parameters were used in molecular docking simulations performed with AutoDock Vina 1.1.2 (Trott and Olson, 2010). For each compound, the top 20 binding conformations were recorded. The resulting docking poses were visually inspected using PyMOL™ 1.7.5.4 Edu (Schrödinger, LLC, New York, NY, United States).

### 2.8 ADME properties

The Administration, Distribution, Metabolism, and Excretion (ADME) properties of the secondary metabolites assessed on tyrosinase were calculated using the SwissADME platform (Daina et al., 2017). The parameters analyzed included the number of atoms with hydrogen-bond acceptor capacity (H-bond acceptors), molar refractivity (MR), octanol/water partition coefficient estimated from solvation free energy in implicit solvents (iLogP), and aqueous solubility predicted directly from molecular structure (ESOL log S), along with the corresponding solubility classification (ESOL class). Additional descriptors comprised predicted gastrointestinal absorption (GI absorption), blood–brain barrier permeability (BBB permeant), and the probability of acting as a P-glycoprotein substrate (P-gp substrate). Furthermore, cytochrome P450 isoenzyme inhibition profiles (CYP_2C19_, CYP_2C9_, CYP_2D6_, and CYP_3A4_), the number of Lipinski’s rule-of-five violations, and the predicted oral bioavailability according to the Abbott score were also considered.

### 2.9 PAINS and brenk alert assessment

Structural alerts associated with PAN-assay interference compounds (PAINS) and medicinal chemistry liabilities (Brenk filters) were evaluated individually for each compound using SwissADME (accessed February 2026).

### 2.10 Cellular studies

Human epidermal melanocytes derived from darkly pigmented donors (HEMs-DP) were used from human origin and purchased as preserved cryovials from Invitrogen is part of Thermo Fisher Scientific (Waltham, MA, United States; Cat No. C2025C). Each cell batch was tested negative for HIV-1, HIV-2, HCV, and other antigens by the manufacturer. Cell cultures were initiated following the manufacturer’s suggested protocols using Human Melanocyte Growth Supplement (HMGS)-supplemented Medium 254 Gibco of Thermo Fisher Scientific (Waltham, MA, United States; Cat No. S-002–5). Complete supplemented medium contained 0.2% v/v bovine pituitary extract (BPE), 0.5% v/v fetal bovine serum, 5 μg/mL bovine insulin, 5 μg/mL bovine transferrin (5 μg/mL), 3 ng/mL basic fibroblast growth factor, 0.18 μg/mL hydrocortisone, 3 μg/mL heparin, and 10 ng/mL phorbol 12-myristate 13-acetate (PMA). HEMs-DP cells were seeded in 24-well plates at a density of 3.3 × 10^4^ cells/500 µL per well (*n* = 6 wells per treatment group). The experimental setup included four conditions: untreated control, vehicle control (1% v/v DMSO), and test compound treatments at 40 ppm and 250 ppm (µg/mL). Treatments were administered *in vitro* directly to the cell culture medium on day 1 (Makpol et al., 2009).

### 2.11 Tissues

MatTek’s MelanoDerm™ tissue inserts, derived from darkly pigmented donors (Cat. No. MEL-300-B), were used to culture human-derived epidermal keratinocytes (NHEK) and melanocytes (NHM), which were then grown on cell culture inserts at the air-liquid interface to form a multilayered, highly differentiated model of the human epidermis. Cells were tested negative for HIV-1, HIV-2, HCV, and other antigens by the manufacturer. Tissue culture was initiated according to the manufacturer’s suggested protocols, using maintenance medium (EPI-100-NMM-113). Cells were cultured according to the MatTek Corp. protocol for the MEL-300-B model, acclimated for 1 h in Medium EPI-100-NMM-113 before dosing, and cell groups were identified using appropriate cell culture plates (Kim et al., 2012).

### 2.12 Environmental conditions

The cell culture incubator was set to a constant 5% CO_2_ in air, with temperature and humidity monitored and controlled, maintained at 36 °C–38 °C and 93%–96%, respectively.

### 2.13 Cell viability assay

Cell viability was assessed using the CellTiter 96® AQueous One Solution Cell Proliferation Assay Promega (Madison, WI, United States), a colorimetric method based on the bioreduction of the tetrazolium compound MTS into a soluble formazan product by metabolically active cells. After treatment, 20 µL of CellTiter 96® AQueous One Solution Reagent was added directly to each well of the culture plates and incubated for 30–60 min at 37 °C in a humidified 5% CO_2_ atmosphere, protected from light. The absorbance was measured at 490 nm using a 96-well microplate reader, and the signal, proportional to the number of viable cells, was expressed as a percentage of vehicle-treated controls (Barltrop et al., 1991).

### 2.14 Melanin content in cell lysates

Melanin content in cell monolayers was quantified using an alkali-solubilization colorimetric assay with synthetic melanin as a standard. Briefly, after treatments, cells were washed with PBS, harvested, and lysed in 1 N NaOH (200–250 µL per well), then incubated at 60 °C–80 °C for 1–2 h to ensure complete melanin solubilization. The absorbance of the lysates was measured at 450 nm in a 96-well plate reader, and melanin concentrations were interpolated from a standard curve prepared with serial dilutions of synthetic melanin dissolved in 1 N NaOH (Supplementary Figure S9) (Makpol et al., 2009).

### 2.15 Melanin content in 3D skin equivalents

Melanin content in human skin equivalents (MelanoDerm™) was determined using a Solvable™-based melanin assay adapted from the manufacturer’s protocol. After treatment, tissue inserts were immersed in PBS for at least 10 min to remove residual phenol red and test article, filled with 300 µL of 1% (w/v) NaHCO_3,_ and the tissues were then excised, gently blotted dry, and transferred to 1.7-mL microcentrifuge tubes. Each tissue was incubated overnight at 60 °C with 250 µL of Solvable™ Tissue Solubilizer (Packard BioScience Co., currently marketed by Revvity/PerkinElmer, CT, United States; Cat. No. 6NE9100).

Together with synthetic melanin standards prepared in Solvable™ (stock 1,000 μg/mL, followed by two-fold serial dilutions to obtain 4–500 μg/mL), then vortexed, cooled to room temperature, centrifuged at 13,000 rpm for 5 min, and 100 µL of each supernatant was transferred to a 96-well plate for absorbance measurement at 490 nm. Melanin content per tissue was calculated from the standard curve and normalized to tissue area or protein content, as appropriate.

### 2.16 Statistical analysis

Statistical analyses, including graph generation, calculation of kinetic constants, and Dunnett’s multiple comparisons test, were performed using GraphPad Prism version 8.0 (Inc., La Jolla, CA, United States).

## 3 Results and discussion

Aporphine alkaloids and some of their derivatives have shown remarkable antioxidant activity *in vitro*, demonstrating a high capacity to deactivate stable free radicals, such as DPPH through hydrogen atom transfer mechanism (HAT), and reduce Fe(III) to Fe(II) by single electron transfer mechanism (SET), even surpassing the synthetic antioxidant BHT (Mellado et al., 2015). In this context, BOL has stood out as one of the most active compounds, exhibiting values between 16 and 33 μg/mL in different radical assays, including ABTS, superoxide, hydroxyl, and even hydrogen peroxide radicals, showing performance similar to or better than that of reference antioxidants (Jaganathan et al., 2025). Additionally, 3BrBOL was evaluated for its previously reported potent antioxidant activity, which inhibits ROS production in f-MLP-stimulated human neutrophils (IC_50_ = 0.81 μM) and the hypoxanthine–xanthine oxidase system (IC_50_ = 0.55 μM) (Milián et al., 2012). Given their antioxidant activity and the known oxidative activity of tyrosinase (Joo et al., 2025), the ability of these compounds to inhibit the enzyme was evaluated. The IC_50_ values, Michaelis constants (K_m_), and maximum velocities (V_max_) were determined similarly for each of the isolated pure alkaloids. All these results are shown in Table 1.

**TABLE 1:** Tyrosinase inhibition values for the alkaloids of *Peumus boldus, Cryptocarya alba, L. sempervirens,* boldine derivatives, and the standard inhibitors arbutin, gallic acid, and kojic acid.

Inhibitor	Acronym	IC_50_ (mM)^a^	IC_50_ (μg/mL)^a^	K_m_ (mM)^a^	V_max_ (mM/min)^a^
(*R,S*)-coclaurine	CC	1.29	368	1.75	0.07
(*R,S*)-*N*-methylcoclaurine	NMCC	1.46	437	1.10	0.04
Reticuline	RET	4.71	1,554	0.45	0.04
Laurolitsine	LTS	0.96	301	0.99	0.08
Boldine	BOL	6.56	2,145	0.33	0.03
Laurotetanine	LTT	3.38	1,105	0.44	0.04
*N*-methyllaurotetanine	NMLT	2.23	760	3.58	0.11
Isocorydine	ISO	3.93	1,340	0.77	0.05
Diacetylboldine	DAB	2.22	913	—	—
3-Bromoboldine	3BrBOL	1.96	796	—	—
Arbutin	ARB	0.061	17	—	—
Gallic acid	GA	0.20	34	—	—
Kojic acid	KA	0.014	2	—	—

^a^ At 0.84 mM L-DOPA; (−) not quantified (*n* = 3).

Regarding tyrosinase inhibition, it should be noted that the mushroom enzyme, although different from human tyrosinase involved in melanogenesis, is generally considered a suitable model for preliminary studies (Pillaiyar et al., 2017; Wang et al., 2023). As mentioned in the introduction, BOL was shown several years ago to be a mixed inhibitor of mushroom tyrosinase and has since been considered a potential candidate for inhibiting melanogenesis (Si et al., 2013). In our study of eight alkaloids from native Chilean species, BOL was the least potent, with an IC_50_ of 6.56 mM, which is virtually identical to the published value (Si et al., 2013). LTS, the *N*-demethylated analog of BOL, is the most potent compound in our collection, with an IC_50_ of 0.96 mM, more than six times that of BOL. Other relatively potent constituents include the benzylisoquinolines CC and NMCC; however, their IC_50_ values remain higher than those of ARB, GA, and KA, which were used as references.

The inhibitory behavior exhibited by NMCC suggests that this alkaloid competes with the endogenous substrate L-DOPA for the tyrosinase active site. The phenolic structure of NMCC could also make it a substrate for tyrosinase’s monophenolase activity, thereby decreasing the free enzyme available for oxidizing L-DOPA (diphenolase activity) and, consequently, reducing its apparent affinity for the substrate without altering V_max_. It is important to note that the formation of dopaquinone and ultimately dopachrome, due to the enzyme’s diphenolase activity, is limited by the concentration of molecular oxygen at the active site. This condition was not controlled, as the experiments were conducted under atmospheric conditions, implying that the K_m_ values are apparent constants. NMCC was one of 10 alkaloids analyzed from the lotus flower (*Nelumbo nucifera*) for its inhibitory activity on theophylline-stimulated melanogenesis in murine B164A5 melanoma cells, with an IC_50_ of 6.5 µM, making it more active than ARB (Morikawa et al., 2016). In a very similar study, on the antimelanogenic effect of green embryo alkaloids of the lotus seed on murine B16F10 melanoma cells stimulated with α-MSH, it was found that, of five benzylisoquinoline alkaloids evaluated, the most significant decrease in melanin content (∼45% melanin) corresponded to juzifine and norjuzifine—both with a methoxy group at C-8 and a hydroxyl group at C-9—, while NMCC reached about 70% melanin, still better than ARB (0.5 μg/mL) (Chen et al., 2023).

To complement the study of alkaloid inhibition of tyrosinase, a molecular docking analysis was performed using the crystal structure of tyrosinase isolated from *Agaricus bisporus*. The molecular docking protocol was validated, and the crystallized ligand (tropolone) was initially removed from the tyrosinase structure (PDB ID: 2Y9X) and reattached to the same crystallographic conformation. This procedure yielded an affinity energy of −5.7 kcal/mol (Supplementary Figure S10), consistent with the value reported by El-Sharkawy et al. (2024). Once the active site was properly recognized and the dimensions of the evaluated compounds were taken into account, the same protocol was applied to each compound. ARB, GA, and KA were also included as positive controls, with affinity energies of −5.8, −5.5, and −5.4 kcal/mol, respectively. These values fall within the range previously reported for KA, whose experimental affinity energy has been estimated at −4.7 kcal/mol (El-Sharkawy et al., 2024).

Subsequently, molecular docking of the aporphine alkaloids was performed using the coordinates of the previously defined active site (Supplementary Figures S11–S16), yielding affinity energies ranging from −5.3 to −7.3 kcal/mol. These results are summarized in Table 2. The molecular docking results show that all evaluated alkaloids yielded negative docking scores within this energy range, suggesting potential compatibility with the catalytic pocket under the applied docking conditions. Among them, LTS displayed the strongest interaction (−7.3 kcal/mol), followed closely by RET (−6.9 kcal/mol) and NMCC (−6.8 kcal/mol). All compounds interacted primarily with the catalytic residues His259, His263, Asn260, Val283, and Ser282, which are characteristic of the enzyme’s active site. Hydrogen bonding was commonly observed with His263, Asn260, and Ser282, reinforcing their roles in substrate recognition and stabilization, while van der Waals interactions contributed to overall binding stability. In contrast, BOL exhibited the lowest affinity, lacking hydrogen-bonding interactions, although it maintained several hydrophobic contacts with residues near the active site. These observations suggest that structural differences among benzylisoquinoline alkaloids may influence their predicted interaction profiles within the catalytic pocket.

**TABLE 2:** Molecular docking results of the natural compounds on tyrosinase and their interactions with the amino acid residues.

Compound	Affinity energy (kcal/mol)	The interaction type of amino acid residues
(*R,S*)-coclaurine	−6.6	Hydrogen bonding: His263 (3.6Å), Ser282 (3.6 Å) Van der Waals: His259, His263, Val283, Asn260, Phe264, Ser282, Pro277, His85
(*R,S*)-*N*-methylcoclaurine	−6.8	Hydrogen bonding: His263 (3.0 Å), Ser282 (3.6 Å) Van der Waals: His259, His263, Val283, Asn260, Phe264, Pro277, His244, Ser282, Val248
Reticuline	−6.9	Hydrogen bonding: His263 (3.5 Å), Ser282 (2.0 Å) Van der Waals: His259, His263, Val283, Asn260, Phe264, Pro277, Ser282, His244, Val248, His85
Laurolitsine	−7.3	Hydrogen bonding: His259 (3.4 Å), Asn260 (3.2 Å) Van der Waals: His259, His263, Val283, Asn260, Phe264, His244, Ser282, Val248, His85
Boldine	−5.3	Hydrogen bonding: Van der Waals: His259, His263, Val283, Asn260, Phe264, His244, Ser282, Val248, His85, Glu322
Laurotetanine	−5.6	Hydrogen bonding: Asn260 (2.6 Å) Van der Waals: His259, His263, Val283, Asn260, Phe264, Pro277, His244, Val248, His85
*N*-methyllaurotetanine	−5.9	Hydrogen bonding: His263 (2.7 Å) Van der Waals: His259, His263, Val283, Asn260, Phe264, His244, Ser282, Val248, His85
Isocorydine	−6.1	Hydrogen bonding: Asn260 (3.1 Å) Van der Waals: His259, His263, Val283, Asn260, Phe264, His244, Ser282, Val248, His85
3-Bromoboldine	−5.5	Hydrogen bonding: Van der Waals: His259, His263, Val283, Asn260, Phe264, His244, Ser282, Val248, His85
Diacetylboldine	−6.1	Hydrogen bonding: Van der Waals: His259, His263, Val283, Asn260, Phe264, His244, Ser282, Val248, His85, Glu322

Two hemisynthetic alkaloids derived from BOL, 3BrBOL, and DAB, were evaluated as potential tyrosinase inhibitors and included in the molecular docking analysis. Both compounds exhibited affinity energies comparable to those of BOL, with −5.5 kcal/mol for 3BrBOL and −6.1 kcal/mol for DAB. Analysis of interactions at the active site indicates that these derivatives maintain recognition patterns similar to those of the natural compound and interact with the same amino acid residues. However, the increase in the molecular volume of these derivatives seems to slightly favor hydrophobic contacts within the catalytic pocket.

It should be noted that DAB has been incorporated into depigmenting formulations due to its inhibitory activity on tyrosinase (Mas-Chamberlin et al., 2004); however, the affinity energies obtained in this study do not exceed those of positive controls, such as ARB, GA, and KA. This apparent discrepancy between the *in silico* scoring and its reported application may reflect limitations of docking approaches, which do not account for solvation, protein flexibility, or pharmacokinetic factors.

**FIGURE 2:**
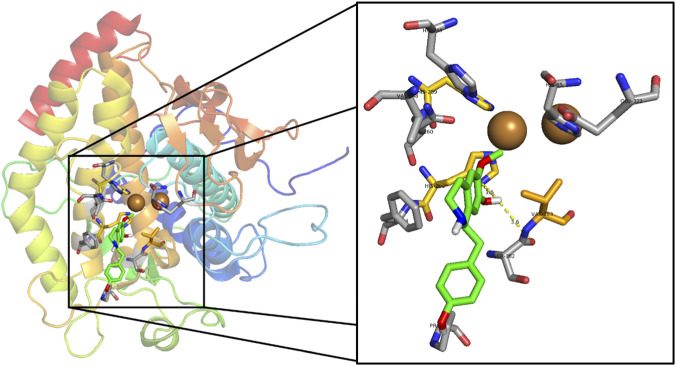
Visualization of molecular docking results for the alkaloid coclaurine and scale-up within the active site of the tyrosinase enzyme (PDB ID: 2Y9X). Color code: Green: carbon atoms for the docked alkaloid. Yellow: carbon atoms of the amino acids being to the active site. Grey: carbon atoms that are close to the active site and participate in the stabilization of the natural alkaloid. Red: oxygen atoms. Blue: nitrogen atoms. White: polar hydrogen atom. Dashed yellow line: hydrogen bonding between ligand and amino acid from the active site.

**FIGURE 3:**
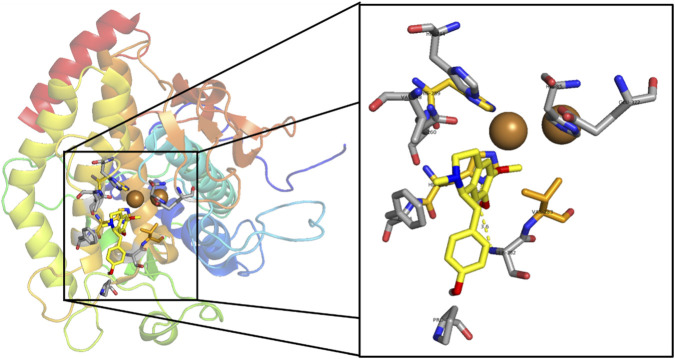
Visualization of molecular docking results for the alkaloid *N*-methylcoclaurine and scale-up within the active site of the tyrosinase enzyme (PDB ID: 2Y9X). Color code: Light yellow: carbon atoms for the docked alkaloid. Yellow: carbon atoms of the amino acids being to the active site. Grey: carbon atoms that are close to the active site and participate in the stabilization of the natural alkaloid. Red: oxygen atoms. Blue: nitrogen atoms. White: polar hydrogen atom. Dashed yellow line: hydrogen bonding between ligand and amino acid from the active site.

Among the benzylisoquinoline alkaloids evaluated, NMCC showed a higher affinity for tyrosinase (−6.8 kcal/mol) than CC (−6.6 kcal/mol). Both compounds form key hydrogen bonds with His263 and Ser282, although NMCC exhibits a stronger interaction with His263 (3.0 Å vs. 3.6 Å). Van der Waals interactions involve similar hydrophobic residues (His259, His263, Val283, Asn260, Phe264, Ser282, and Pro277), with NMCC extending additional contacts to His244 and Val248, which could explain its slight superiority in affinity (Figures 2, 3). In molecular modeling against tyrosinase (human homology model), the highest affinity was found for 16 alkaloids of *Glaucium acutidentatum* Hausskn. and Bornm. [Papaveraceae] (lotusine, isoboldine/BOL, NMCC, 1,2-dehydroreticuline, reticuline, 4′-*O*-methyl-*N*-methylcoclaurine, isocorydine, laudanine, corydine, 3-hydroxyglaucine, protopine, allocryptopine, glaucine, cataline, and corunnine), 1,2-dehydroreticuline obtained the best bond energy score (∼−6 kcal/mol) among the alkaloids evaluated, *N*-methylcoclaurine exhibited good, albeit inferior, binding affinity, while its derivative 4-*O*-methyl-*N*-methylcoclaurine showed specific interactions at the active site: hydrogen bonds, cation-π, sigma, π-π stacking, van der Waals forces and contact with catalytic Cu^2+^ ions (Yagi et al., 2024). In another molecular modeling using tyrosinase (human homology model, Surflex-Dock), the alkaloids of *L. aggregata* [Lauraceae] showed the highest affinity: (−)-*N*-methyllaurotetanine (score −4.30), reticuline (−4.25), laurolitsine/norboldine (−4.10), pallidine (−4.05), and boldine (−1.92), outperforming arbutin (−3.87) and kojic acid (−3.84). (−)-*N*-methyllaurotetanine obtained the best score among the alkaloids evaluated, with key interactions at the active site (H bonds with Glu203, Asp317, Ser361, Val484, Ala490; van der Waals forces and π contacts), while reticuline and laurolitsine exhibited strong affinities with a good skin permeability profile (Fong and Tong, 2012), very similar to our results in mushroom tyrosinase.

Among the compounds evaluated, LTS was the most active inhibitor, with an affinity of −7.3 kcal/mol. In contrast, BOL, the least active compound within this series of natural compounds, exhibited the highest affinity energy (−5.3 kcal/mol). Although docking scores cannot be directly equated with experimental affinity, a qualitative agreement between predicted binding energies and inhibitory potency was observed for some compounds.

**FIGURE 4:**
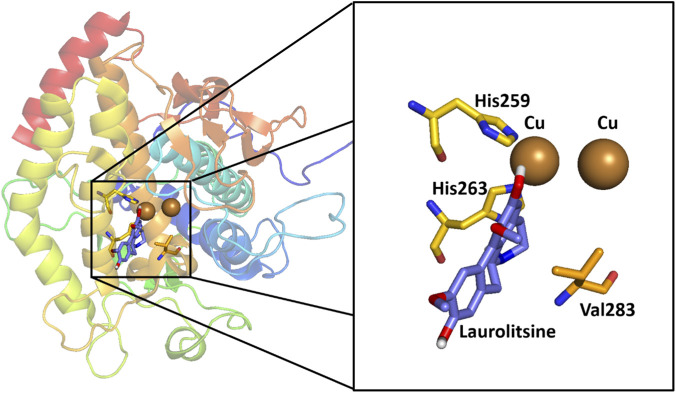
Visualization of molecular docking results for the alkaloid laurolitsine and scale-up within the active site of the tyrosinase enzyme (PDB ID: 2Y9X).

**FIGURE 5:**
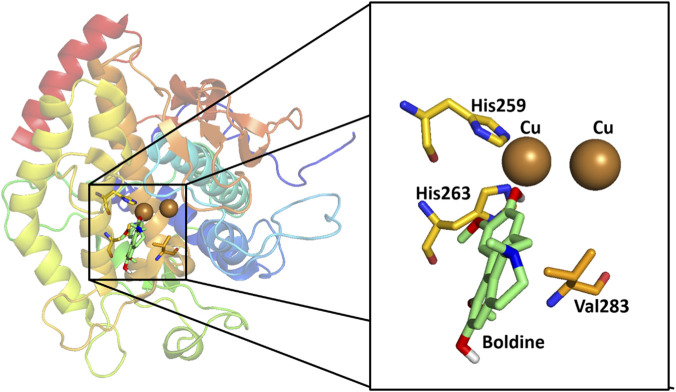
Visualization of molecular docking results for the alkaloid boldine and scale-up within the active site of the tyrosinase enzyme (PDB ID: 2Y9X).

Structural analysis of the active site revealed that both LTS and BOL direct a hydroxyl group toward residue His259—similar to what is observed with tropolone—with interaction distances of 4.5 Å and 4.2 Å, respectively, between the hydrogen atom and the amino acid residue. However, LTS also exhibits an additional hydrogen-bond interaction between its–NH group and the residue His263 (3.4 Å), absent in BOL because this group is–*N*-methylated. This additional interaction may contribute to the more favorable docking score observed for laurolitsine and is consistent with its higher inhibitory potency; however, this interpretation remains hypothetical and would require further biophysical validation (Figures 4, 5). *N*-Formylanonaine, an aporphine alkaloid from *Magnolia × alba* (DC.) Figlar [Magnoliaceae] (syn. *Michelia alba*) shares structural and functional similarities with LTS and BOL in inhibiting human tyrosinase (IC_50_ = 74.3 µM) —comparable to KA (IC_50_ = 69.4) — through coordination with Cu^2+^ ions at the active site, as confirmed by molecular docking (Wang et al., 2010). Like LTS, it exhibits key hydrophobic interactions with residues such as His259, His263, and Val283; its unmethylated–NH group promotes greater affinity. BOL derivatives have recently been developed by modification at the C-3 position (amidomethylation followed by acylation with hydroxycinnamic acids), dramatically improving the inhibition of mushroom tyrosinase. Boldine caffeoylamide (CafA-Boldine) showed the greatest potency (superior to hydroquinone) due to its catechol group, similar to that of L-DOPA, whereas feruloylamide (FA-Boldine) was equipotent to HQ. These results demonstrate that specific C-3 substitutions enhance the inhibitory activity of the BOL scaffold, suggesting promising structural optimizations for aporphine alkaloid derivatives in cosmetics (Chochkova et al., 2024).

Overall, the docking results should be interpreted as structural hypotheses that are consistent with the enzymatic inhibition data but do not constitute direct evidence of binding affinity or mechanistic specificity. Further kinetic, biophysical, or molecular dynamics studies would be required to confirm the proposed binding modes.

In addition to activity and docking analyses, structural alerts related to assay interference and medicinal chemistry liabilities were assessed to better understand the pharmacological significance of these compounds. No PAINS alerts were found for any of the alkaloids examined. Only diacetylboldine (DAB) triggered a Brenk alert because of its ester group (Supplementary Table S4). The lack of PAINS alerts suggests that the tyrosinase inhibition observed is unlikely to result from known assay-interfering substructures.

Since BOL is a commercially used compound, it was selected along with two of its derivatives —3BrBOL and DAB—for an exploratory study aimed at evaluating the depigmenting potential of aporphine alkaloids.

**FIGURE 6:**
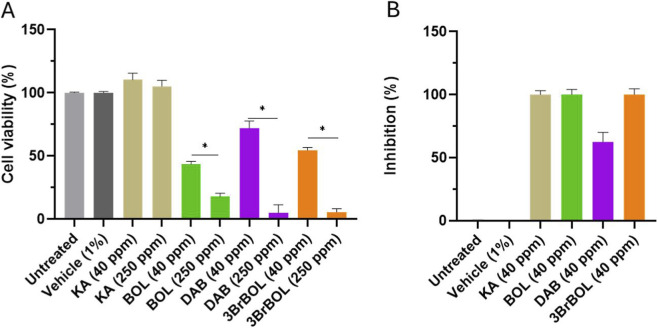
**(A)** Cell viability results after 24-h treatments of test and control materials in cultured HEMs-DP cells (*n* = 6). Statistical significance was determined by one-way ANOVA followed by Dunnett’s *post hoc* test (*P < 0.05). **(B)** Melanin content in cell lysates in cultured HEMs-DP cells (*n* = 6).

To determine the optimal assay concentration, cell viability was evaluated at 40 ppm and 250 ppm, with greater viability observed at the lower concentration. This concentration corresponds to the final DAB level (40 ppm or 40 μg/g) recommended by the manufacturer, based on clinical trials and derived from *in vitro* results (Mas-Chamberlin et al., 2004). Therefore, this concentration was selected to evaluate both cell viability and melanin production in melanocytes (Figure 6A).

All three compounds reduced cell viability compared to cells treated with the vehicle alone. This unexpected result suggests that lower concentrations should be used in subsequent studies to better distinguish potential cytotoxic effects from those directly associated with melanogenesis inhibition.

Nevertheless, BOL and 3BrBOL inhibited tyrosinase activity by 100% at 40 ppm (single dose), outperforming DAB. As a positive control, KA showed a greater decrease in melanin content at 40 ppm relative to untreated or vehicle-treated cells after 24 h of incubation. Among the aporphine compounds evaluated, 3BrBOL and BOL produced the most marked inhibition of melanin (100%). In contrast, DAB—a currently commercially available compound—achieved 62% inhibition in human melanocytes (HEMs-DP) at the same concentration (40 ppm, single dose) (Figure 6B).

**FIGURE 7:**
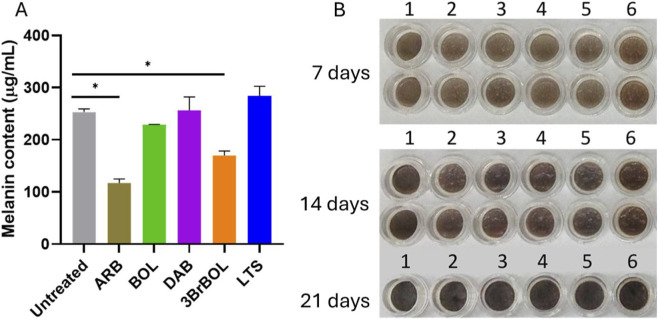
**(A)** Melanin content in cell lysates after 14 days of treatment with test or control materials in MelanoDerm™ (*n* = 3). Statistical significance was determined by one-way ANOVA followed by Dunnett’s *post hoc* test (*P < 0.05). **(B)** Representative images of MelanoDerm™ tissues after 14 days of treatment: 1) Untreated (negative control); 2) ARB (0.1% w/v, positive control); 3) DAB (0.1% w/v); 4) 3BrBOL (0.1% w/v); 5) BOL (0.1% w/v); 6) LTS (0.1% w/v).

After 14 days of incubation, MelanoDerm™ tissues treated with a single dose of 0.1% of ARB and 3BrBOL showed a significant decrease in melanin content. The reduction with 3BrBOL was 33% relative to untreated tissues. This level of inhibition suggests that 3BrBOL exerts a depigmenting effect, albeit less pronounced than that of the reference agent, ARB, which is widely used in cosmetic formulations to control hyperpigmentation (Doğan and Akocak, 2024). In contrast, boldine induced only a slight decrease in melanin content (10%). At the same time, DAB had no effect, and LTS slightly increased melanin, demonstrating that modifications in the aporphine nucleus directly modulate melanogenic inhibition (Figure 7).

Tyrosinase inhibition is a primary mechanism for reducing melanogenesis; however, translating this enzymatic activity into cellular models depends on additional factors, including cell permeability, metabolic stability, and regulation of melanogenic pathways. In this context, the absorption, distribution, metabolism, and excretion (ADME) properties of the studied compounds and positive controls were evaluated using the SwissADME platform to estimate their pharmacokinetic behavior.

The results indicate that both alkaloids and the positive controls exhibit comparable physicochemical properties, including molecular weight, number of rotatable bonds, and hydrogen bond donor and acceptor capacities (Supplementary Table S5). Nevertheless, significant differences were observed in parameters associated with lipophilicity, including the iLogP index and polar surface area (TPSA). Specifically, alkaloids exhibited higher iLogP values and lower TPSA values, suggesting a more lipophilic character compared with the positive controls, which showed a more hydrophilic profile, also reflected by higher aqueous solubility (LogS) values.

Consistent with these findings, the estimated skin permeability (logKp) values for the natural alkaloids were approximately −6.3 cm/s, whereas the positive controls exhibited an average of −7.8 cm/s. These differences suggest a greater capacity for passive skin permeation for alkaloids, which may help explain why, despite showing weaker direct tyrosinase inhibition *in vitro*, these compounds display antimelanogenic activity comparable to that of positive controls such as ARB and KA in cellular models (Figures 6, 7).

**FIGURE 8:**
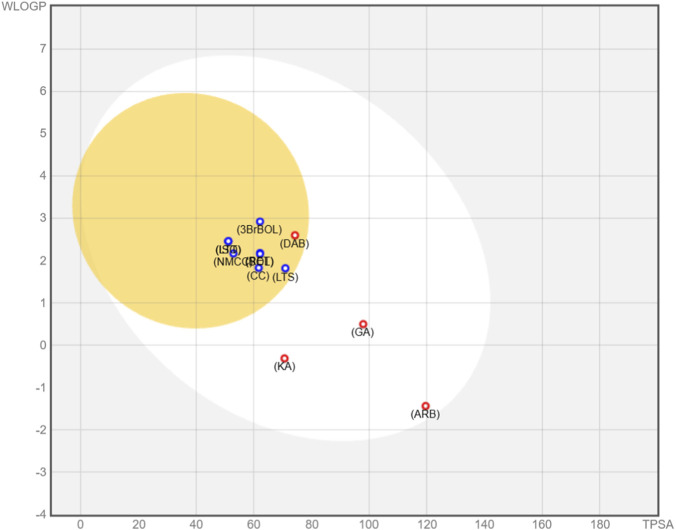
Boiled egg scheme for alkaloids and positive controls used in tyrosinase inhibition assay. Blue circles: P-gp substrate. Red circles: Non-P-gp substrate. CC: (*RS*)-Coclaurine; NMCC: (*RS*)-*N*-methylcoclaurine; RET: Reticuline; LTS: Laurolitsine; BOL: Boldine; LTT: Laurotetanine; ISO: Isocorydine; DAB: Diacetylboldine; 3BrBOL: 3-Bromoboldine, GA: Gallic acid, ARB: Arbutin; KA: Kojic acid.

This trend is further supported by the boiled egg model (Figure 8), in which alkaloids are preferentially located within the lipophilic region (yolk), while positive controls are mainly distributed in the hydrophilic region (white), reinforcing the differences observed in their permeability profiles and potential cellular bioavailability.

## 4 Conclusion

Aporphine alkaloids exhibit potent antioxidant activity *in vitro*, exceeding the oxidative activity of tyrosinase, thereby justifying their evaluation as inhibitors. LTS exhibited greater enzymatic inhibition of mushroom tyrosinase than BOL, with a potency six times greater. This effect can be explained by the hydrogen bonding of the–NH fragment, as shown in the molecular docking analysis. In addition, comparing the benzylisoquinoline and aporphine cores, both nuclei contribute to the stabilization of the alkaloids through van der Waals interactions. These findings extend the evidence for depigmenting aporphines: BOL acts as a mixed tyrosinase inhibitor. NMCC is a competitive inhibitor of mushroom tyrosinase, making benzylisoquinolines promising and warranting further investigation.

In HEMs-DP melanocytes, BOL and 3BrBOL completely inhibited tyrosinase, demonstrating superior performance compared with the commercial reference compound (62% DAB at 40 ppm), although cytotoxicity requires optimization. In MelanoDerm™, 3-bromoboldine reduced melanogenesis by 33%, comparable to ARB (33%) and exceeding BOL (10%), whereas DAB was inactive. Therefore, halogenation at the three-position of boldine enhances this activity in 3D models, surpassing that of natural BOL. This suggests that strategic halogenation improves interaction with tyrosinase in human melanocytes, justifying its development as a cosmetic candidate or, given its ease of synthesis, as a precursor to other depigmenting agents derived from BOL. Future formulations (such as nanoemulsions, as in DAB) could enhance skin retention and increase potency, positioning halogenated derivatives as high-quality sparing agents for hyperpigmentation and facilitating the clinical translation of alkaloids from the Chilean flora.

## Data Availability

The original contributions presented in the study are included in the article/Supplementary Material, further inquiries can be directed to the corresponding author.

## References

[B1] A Okoye G. Bui H. Zadu A. Myles I. A. S Byrd A. (2025). The multifaceted effects of berberine: potential uses in dermatology. J. Drugs Dermatol. 24, 298–301. 10.36849/JDD.8899 40043268

[B2] Abouelela M. Naimy R. Elshafey O. Mahmoud M. Elgez M. Ahmed M. (2023). Boldo phytochemical and pharmacological activities updated Mini-review. ERU Res. J. 2, 308–319. 10.21608/erurj.2023.203801.1018

[B3] Akalın G. Selamoglu Z. (2019). Nutrition and foods for skin health. J. Pharm. Care 7, 31–33. 10.18502/jpc.v7i1-2.1620

[B4] Al Saqr A. Annaji M. Poudel I. Aldawsari M. F. Alrbyawi H. Mita N. (2023). Topical delivery of diacetyl boldine in a microemulsion formulation for chemoprotection against melanoma. Pharmaceutics 15, 901. 10.3390/pharmaceutics15030901 36986762 PMC10054442

[B5] Al-ghazzawi A. M. (2019). Anti-cancer activity of new benzyl isoquinoline alkaloid from Saudi plant Annona squamosa. BMC Chem. 13, 13. 10.1186/s13065-019-0536-4 31384762 PMC6661725

[B6] Alves R. Castro Esteves T. Trelles M. A. (2013). Factores intrínsecos y extrínsecos implicados en el envejecimiento cutáneo. Cirugía Plástica Ibero-Latinoamericana 39, 89–102. 10.4321/S0376-78922013000100013

[B7] Araya O. Núñez M. Mellado M. Olea A. F. Espinoza-Catalán L. (2025). Synthesis of new brassinosteroid analogs with androstane skeleton and heterocyclic acyl side chains: preliminary molecular docking studies. Molecules 30, 4011. 10.3390/molecules30194011 41097431 PMC12526290

[B8] Barltrop J. A. Owen T. C. Cory A. H. Cory J. G. (1991). 5-(3-carboxymethoxyphenyl)-2-(4,5-dimethylthiazolyl)-3-(4-sulfophenyl)tetrazolium, inner salt (MTS) and related analogs of 3-(4,5-dimethylthiazolyl)-2,5-diphenyltetrazolium bromide (MTT) reducing to purple water-soluble formazans as cell-viability indicat. Bioorg. Med. Chem. Lett. 1, 611–614. 10.1016/S0960-894X(01)81162-8

[B9] Carimonei C. (2024). Valoración del Laurel (*Laurelia sempervirens*) en un entorno amenazado por la intervención humana y el cambio climático. Rev. Nothofagus 9, 101–110.

[B10] Cassels B. K. Fuentes-Barros G. Castro-Saavedra S. (2019). Boldo, its secondary metabolites and their derivatives. Curr. Tradit. Med. 5, 31–65. 10.2174/2215083804666181113112928

[B11] Cassels B. K. Castro-Saavedra S. Fuentes-Barros G. (2021). “Boldine,” in A centum of valuable plant bioactives (Lahora, Pakistan: Elsevier), 491–508.

[B12] Castro-Saavedra S. Fuentes-Barros G. Tirapegui C. Acevedo-Fuentes Wi. Cassels B. K. Barriga A. (2016). Phytochemical analysis of alkaloids from the Chilean endemic tree *Cryptocarya alba* . J. Chil. Chem. Soc. 61, 3076–3080. 10.4067/S0717-97072016000300014

[B13] Chen Y.-C. Liu Y.-Y. Chen L. Tang D.-M. Zhao Y. Luo X.-D. (2023). Antimelanogenic effect of isoquinoline alkaloids from *Plumula nelumbinis* . J. Agric. Food Chem. 71, 16090–16101. 10.1021/acs.jafc.3c03784 37856847

[B14] Chochkova M. Stoykova B. Nikolaeva-Glomb L. Philipov S. Milkova T. (2020). Synthesis of 3-aminomethylglaucine derivatives and *in vitro* evaluation of their anti-tyrosinase, antiviral and radical scavenging activities. J. Chem. Technol. Metall. 55, 261–271.

[B15] Chochkova M. Stoykova B. Petrova P. Štícha M. Ivanova G. (2024). Synthesis of novel boldine amides and their *in vitro* inhibitory effects on a mushroom tyrosinase. J. Chem. Technol. Metall. 59, 855–862. 10.59957/jctm.v59.i4.2024.14

[B16] Daina A. Michielin O. Zoete V. (2017). SwissADME: a free web tool to evaluate pharmacokinetics, drug-likeness and medicinal chemistry friendliness of small molecules. Sci. Rep. 7, 42717. 10.1038/srep42717 28256516 PMC5335600

[B17] Doğan A. Akocak S. (2024). “Natural products as tyrosinase inhibitors,” in The enzymes, (Firenze, Italy: Universita degli Studi di Firenze) 85–109. 10.1016/bs.enz.2024.06.002 39304292

[B18] El-Sharkawy R. M. El-Hadary A. E. Essawy H. S. El-Sayed A. S. A. (2024). Rutin of Moringa oleifera as a potential inhibitor to *Agaricus bisporus* tyrosinase as revealed from the molecular dynamics of inhibition. Sci. Rep. 14, 20131. 10.1038/s41598-024-69451-y 39209920 PMC11362471

[B19] Fernández J. Lagos P. Rivera P. Zamorano‐Ponce E. (2009). Effect of boldo (*Peumus boldus* Molina) infusion on lipoperoxidation induced by cisplatin in mice liver. Phyther. Res. 23, 1024–1027. 10.1002/ptr.2746 19145575

[B20] Figueiredo M. B. G. A. Santana V. R. de Nardelli M. J. Nogueira M. de S. Azevedo D. X. Santana D. P. A. (2016). The effect of the aqueous extract *Peumus boldus* on the proliferation of hepatocytes and liver function in rats submitted to expanded hepatectomy. Acta Cir. Bras. 31, 608–614. 10.1590/S0102-865020160090000006 27737346

[B21] Fisher G. J. Kang S. Varani J. Bata-Csorgo Z. Wan Y. Datta S. (2002). Mechanisms of photoaging and chronological skin aging. Arch. Dermatol. 138, 1462–1470. 10.1001/archderm.138.11.1462 12437452

[B22] Fong P. Tong H. H. Y. (2012). *In silico* prediction of the cosmetic whitening effects of naturally occurring lead compounds. Nat. Prod. Commun. 7, 1287–1294. 10.1177/1934578X1200701010 23156992

[B23] Fuentes-Barros G. Castro-Saavedra S. Liberona L. Acevedo-Fuentes W. Tirapegui C. Mattar C. (2018). Variation of the alkaloid content of *Peumus boldus* (boldo). Fitoterapia 127, 179–185. 10.1016/j.fitote.2018.02.020 29454020

[B24] Fuentes-Barros G. Echeverría J. Mattar C. Liberona L. Giordano A. Suárez-Rozas C. (2023). Phytochemical variation of wild and farmed populations of boldo (*Peumus boldus* Molina). J. Appl. Res. Med. Aromat. Plants 35, 100502. 10.1016/j.jarmap.2023.100502

[B25] Fuentes-Barros G. Castro-Saavedra S. Montalva N. Mellado M. Diaz-Valdés A. Guerrero-Rodríguez C. (2025). *Cryptocarya alba* (Peumo): an endemic Chilean tree with phytochemicals with bioactive potential. Front. Pharmacol. 16, 1665897. 10.3389/fphar.2025.1665897 41415580 PMC12709134

[B26] Gajardo R. (1995). La vegetación natural de Chile: clasificación y distribución geográfica. Santiago, Chile: Editorial Universitaria.

[B27] Giordano A. Fuentes-Barros G. Castro-Saavedra S. González-Cooper A. Suárez-Rozas C. Salas-Norambuena J. (2019). Variation of wecondary metabolites in the aerial biomass of *Cryptocarya alba* . Nat. Prod. Commun. 14, 1934578X1985625. 10.1177/1934578X19856258

[B28] Gotteland M. Espinoza J. Speisky H. (1995). Efecto de un extracto seco de boldo sobre el tránsito intestinal oro-cecal en voluntarios sanos. Rev. Med. Chil. 123, 955–960. 8657963

[B29] Guinaudeau H. Leboeuf M. Cave A. (1975). Aporphine alkaloids. Lloydia 38, 275–338. 241890

[B30] Hara H. Kaneko K. Endoh M. Uchida H. Hoshino O. (1995). A novel ring cleavage and recyclization of N-cyanomethyl-1,2,3,4-tetrahydroisoquinolinium methiodides: a biomimetic synthesis of litebamine. Tetrahedron 51, 10189–10204. 10.1016/0040-4020(95)00614-E

[B31] Ismaya W. T. Rozeboom H. J. Weijn A. Mes J. J. Fusetti F. Wichers H. J. (2011). Crystal structure of *Agaricus bisporus* mushroom tyrosinase: identity of the tetramer subunits and interaction with tropolone. Biochemistry 50, 5477–5486. 10.1021/bi200395t 21598903

[B32] Jaganathan M. Kathiresan S. Muthusamy R. Azhamuthu T. Asath N. A. A. Ravichandran P. (2025). Boldine as a potent anticancer agent: induction of oxidative stress, mitochondrial dysfunction, and apoptosis *via* inhibition of notch signaling in human oral carcinoma cells. J. Biochem. Mol. Toxicol. 39, e70424. 10.1002/jbt.70424 40760846

[B33] Joo Y. Seo Y. H. Lee S. Shin E. Yeon S. W. Kim S. B. (2025). Antioxidant and tyrosinase-inhibitory activities and biological bioactivities of flavonoid derivatives from *Quercus mongolica* pollen. Molecules 30, 794. 10.3390/molecules30040794 40005106 PMC11858624

[B34] Khmaladze I. Leonardi M. Fabre S. Messaraa C. Mavon A. (2020a). The skin interactome: a holistic “Genome-Microbiome-Exposome” approach to understand and modulate skin health and aging. Clin. Cosmet. Investig. Dermatol. 13, 1021–1040. 10.2147/CCID.S239367 33380819 PMC7769076

[B35] Khmaladze I. Österlund C. Smiljanic S. Hrapovic N. Lafon‐Kolb V. Amini N. (2020b). A novel multifunctional skin care formulation with a unique blend of antipollution, brightening and antiaging active complexes. J. Cosmet. Dermatol. 19, 1415–1425. 10.1111/jocd.13176 31584241

[B36] Kim H. Choi H.-R. Kim D.-S. Park K.-C. (2012). Topical hypopigmenting agents for pigmentary disorders and their mechanisms of action. Ann. Dermatol. 24, 1–6. 10.5021/ad.2012.24.1.1 22363147 PMC3283838

[B37] Kubo I. Kinst-Hori I. (1998). Tyrosinase inhibitors from anise oil. J. Agric. Food Chem. 46, 1268–1271. 10.1021/jf9708958

[B38] Lee Y. Liu L. Yi Y. (2018). Inhibitory effect of berberine from *Coptidis rhizoma* on melanin synthesis of murine malignant melanoma. Pharmazie 73, 300–303. 10.1691/ph.2018.7953 29724298

[B39] Luebert F. Pliscoff P. (2006). Sinopsis bioclimática y vegetacional de Chile. Santiago, Chile: Editorial Universitaria.

[B40] Makpol S. Arifin N. N. M. Ismail Z. Chua K. H. Yusof Y. A. M. Ngah W. Z. W. (2009). Modulation of melanin synthesis and its gene expression in skin melanocytes by palm tocotrienol rich fraction. Afr. J. Biochem. Res. 3, 385–392.

[B41] Mas-Chamberlin C. Peschard O. Leroux R. Mondon P. Lamy F. Lintner K. (2004). Di-acetyl-nor-aporphines: novel molecules and novel mechanism to inhibit melanogenesis. SÖFW-journal 130, 2–10.

[B42] Mellado M. Jara C. Astudillo D. Villena J. Reveco P. G. Thomet F. A. (2015). Oxaliplatin analogues with carboxy derivatives of boldine with enhanced antioxidant activity. Bioinorg. Chem. Appl. 2015, 1–7. 10.1155/2015/920143 25814916 PMC4359857

[B43] Milián L. Ballesteros R. Sanz M. J. Blázquez M. A. (2012). Synthesis and reactive oxygen species scavenging activity of halogenated alkaloids from boldine. Med. Chem. Res. 21, 3133–3139. 10.1007/s00044-011-9844-5

[B44] Morganti P. (2015). The meaning of Nano dimension involving cosmetics: from the lab to an industrial green process. J. Sci. Res. Rep. 4, 79–100. 10.9734/JSRR/2015/11182

[B45] Morikawa T. Kitagawa N. Tanabe G. Ninomiya K. Okugawa S. Motai C. (2016). Quantitative determination of alkaloids in Lotus flower (Flower buds of *Nelumbo nucifera*) and their melanogenesis inhibitory activity. Molecules 21, 930. 10.3390/molecules21070930 27447599 PMC6272935

[B46] Nakamura S. Nakashima S. Tanabe G. Oda Y. Yokota N. Fujimoto K. (2013). Alkaloid constituents from flower buds and leaves of sacred lotus (*Nelumbo nucifera*, Nymphaeaceae) with melanogenesis inhibitory activity in B16 melanoma cells. Bioorg. Med. Chem. 21, 779–787. 10.1016/j.bmc.2012.11.038 23270663

[B47] Peralta M. A. Negro M. F. Aguirre E. N. B. Santi M. D. Ortega M. G. (2025). Antifungal and anti-tyrosinase activities of dalea species extracts: differential biological effects and their correlation with phytochemical content *via* UPLC-MS/MS profiling. Rev. Bras. Farmacogn. 35, 567–574. 10.1007/s43450-025-00641-z

[B48] Pillaiyar T. Manickam M. Namasivayam V. (2017). Skin whitening agents: medicinal chemistry perspective of tyrosinase inhibitors. J. Enzyme Inhib. Med. Chem. 32, 403–425. 10.1080/14756366.2016.1256882 28097901 PMC6010116

[B49] Poljšak B. Dahmane R. (2012). Free radicals and extrinsic skin aging. Dermatol. Res. Pract. 2012, 1–4. 10.1155/2012/135206 22505880 PMC3299230

[B50] Pratchyapurit W. (2016). Combined use of two formulations containing diacetyl boldine, TGF-β1 biomimetic oligopeptide-68 with other hypopigmenting/exfoliating agents and sunscreen provides effective and convenient treatment for facial melasma. Either is equal to or is better than. J. Cosmet. Dermatol. 15, 131–144. 10.1111/jocd.12201 26833454

[B51] Ravichandran S. Selamoglu Z. (2023). Anti-inflammatory influences of royal jelly and melittin and their effectiveness on wound healing. Cent. Asian J. Med. Pharm. Sci. Innov. 3, 38–47. 10.22034/CAJMPSI.2023.02.02

[B52] Ren S. Jin J. Wu X. Han B. Zhang W. Rong F. (2025). Effect of an herbal gel for the prevention of radiation dermatitis-related symptoms: an open-label randomized clinical trial. J. Dermatol. Treat. 36, 2489595. 10.1080/09546634.2025.2489595 40229671

[B53] Schmeda‐Hirschmann G. Loyola J. I. Rodriguez J. Dutra‐Behrens M. (1994). Hypotensive effect of *Laurelia sempervirens* (Monimiaceae) on normotensive rats. Phyther. Res. 8, 49–51. 10.1002/ptr.2650080112

[B54] Si Y.-X. Ji S. Wang W. Fang N.-Y. Jin Q.-X. Park Y.-D. (2013). Effects of boldine on tyrosinase: inhibition kinetics and computational simulation. Process Biochem. 48, 152–161. 10.1016/j.procbio.2012.11.001

[B55] Sobarzo-Sánchez E. M. Arbaoui J. Protais P. Cassels B. K. (2000). Halogenated boldine derivatives with enhanced monoamine receptor selectivity. J. Nat. Prod. 63, 480–484. 10.1021/np990433j 10785418

[B56] Song Y. C. Lee Y. Kim H. M. Hyun M. Y. Lim Y. Y. Song K. Y. (2015). Berberine regulates melanin synthesis by activating PI3K/AKT, ERK and GSK3β in B16F10 melanoma cells. Int. J. Mol. Med. 35, 1011–1016. 10.3892/ijmm.2015.2113 25716948

[B57] Speisky H. Cassels B. K. (1994). Boldo and boldine: an emerging case of natural drug development. Pharmacol. Res. 29, 1–12. 10.1016/1043-6618(94)80093-6 8202440

[B58] Tadokoro T. Bonte F. Archambault J. C. Cauchard J. H. Neveu M. Ozawa K. (2010). Whitening efficacy of plant extracts including orchid extracts on Japanese female skin with melasma and lentigo senilis. J. Dermatol. 37, 522–530. 10.1111/j.1346-8138.2010.00897.x 20536665

[B59] Teitel S. Brossi A. (1968). An improved synthesis of various racemic polyphenolic tetrahydroisoquinoline alkaloids. J. Heterocycl. Chem. 5, 825–829. 10.1002/jhet.5570050614

[B60] Thomet F. A. Pinyol P. Villena J. Espinoza L. J. Reveco P. G. (2010). Cytotoxic thiocarbamate derivatives of boldine. Nat. Prod. Commun. 5, 1587–1590. 10.1177/1934578X1000501015 21121254

[B61] Tobin D. J. (2017). Introduction to skin aging. J. Tissue Viability 26, 37–46. 10.1016/j.jtv.2016.03.002 27020864

[B62] Tomas M. Günal-Köroğlu D. Kamiloglu S. Ozdal T. Capanoglu E. (2025). The state of the art in anti-aging: plant-based phytochemicals for skin care. Immun. Ageing 22, 5. 10.1186/s12979-025-00498-9 39891253 PMC11783858

[B63] Tomita M. Kugo T. (1956). Alkaloids of berberidaceous plants - XIX: alkaloids of B. tschonoskyana I. Isolation of bases. Yakugak Zasshi 79, 317–321.

[B64] Touma J. Navarro M. Sepúlveda B. Pavon A. Corsini G. Fernández K. (2020). The chemical compositions of essential oils derived from *Cryptocarya alba* and *Laurelia sempervirens* possess antioxidant, antibacterial and antitumoral activity potential. Molecules 25, 5600. 10.3390/molecules25235600 33260521 PMC7729746

[B65] Trott O. Olson A. J. (2010). AutoDock Vina: improving the speed and accuracy of docking with a new scoring function, efficient optimization, and multithreading. J. Comput. Chem. 31, 455–461. 10.1002/jcc.21334 19499576 PMC3041641

[B66] Veerichetty V. Saravanabavan I. Ramapalaniappan A. (2023). *In silico* kinase inhibition profiling of BRAF and AKT signaling in melanoma cells with nuciferine. J. Phytopharm. 12, 152–163. 10.31254/phyto.2023.12303

[B67] Wan C. Langyan S. Echeverría J. Devkota H. P. Tewari D. Moosavi M. A. (2023). Edible fruits and berries as a source of functional polyphenols: current scene and future perspectives. Phytochem. Rev. 24, 1197–1225. 10.1007/s11101-023-09892-x

[B72] Wang H. M. Chen C. Y. Chen C. Y. Ho M. L. Chou Y. T. Chang H. C. (2010). (–)‐N‐Formylanonaine from Michelia alba as a human tyrosinase inhibitor and antioxidant. Bioorg. Med. Chem. 18(14), 5241–5247. 10.1016/j.bmc.2010.05.045 20584613

[B68] Wang Y. Xiong B. Xing S. Chen Y. Liao Q. Mo J. (2023). Medicinal prospects of targeting tyrosinase: a feature review. Curr. Med. Chem. 30, 2638–2671. 10.2174/0929867329666220915123714 36111760

[B69] Wong V. (2021). Resurface, retone and reboost: a multi-modality approach for prejuvenation of millennial patients. J. Clin. Exp. Dermatol Res. S 8, 549.

[B70] Yagi S. Zengin G. Uba A. I. Maciejewska-Turska M. Sieniawska E. Świątek Ł. (2024). Exploring chemical composition, antioxidant, enzyme inhibitory and cytotoxic properties of *Glaucium acutidentatum* hausskn. and Bornm. from Turkey flora: a novel source of bioactive agents to design functional applications. Antioxidants 13, 643. 10.3390/antiox13060643 38929082 PMC11200578

[B71] Zouboulis C. C. (2007). Molecular mechanisms of skin aging. Ann. N. Y. Acad. Sci. 1119, 40–50. 10.1196/annals.1404.027 18056953

