# Supplementary material for: Molecular docking and experimental evaluation of natural alkaloids from Chilean flora (Cryptocarya alba, Peumus boldus, and Laurelia sempervirens) for tyrosinase inhibition and depigmenting potential
Source: Front Pharmacol. 2026 Apr 14;17:1795496. doi: 10.3389/fphar.2026.1795496 (PMC13121392; doi:10.3389/fphar.2026.1795496)
Supplement: Supplementary file 1 [file Supplementaryfile1.docx]

**Supporting Information**

**Table 1S.** Characterization of studied alkaloids by mass spectrometry.

| **Alkaloid** | **RT (min)** | **[M+H]^+^ (*m/z*)** | **Main MRM** | **Fragments**  **(rel. int. %)** | **Database** |
| --- | --- | --- | --- | --- | --- |
| Coclaurine | 5.35 | 285.9 | 285.9 → 269.1 | 193.9 (18.0),  209.1 (34.8),  237.0 (28.6),  **269.1 (100)** | HMDB0303359^a^  MSBNK-mFam-MC12_000012^b^  CCMSLIB00005436045^d^  (Castro-Saavedra et al., 2016; Torres-Vega et al., 2020) |
| *N*-Methyl-coclaurine | 5.63 | 299.9 | 299.9 → 269.0 | 192.0 (27.4),  209.1 (20.7),  237.1 (20.0),  **269.0 (100)** | CCMSLIB00005436046^d^  (Castro-Saavedra et al., 2016; Schmidt et al., 2005; Tian et al., 2018; Torres-Vega et al., 2020) |
| Laurolitsine  (Norboldine) | 5.75 | 314.0 | 314.0 → 264.9 | 165.1 (51.1),  237.1 (54.3),  **264.9 (100),**  297.1 (90.2) | HMDB0040974^a^  TOF_alkaloids_pos000382^d^  CCMSLIB00005720164^d^  Alkaloids000469^d^  Alkaloids000468^d^  (Castro-Saavedra et al., 2016; Nikolić et al., 2012; Tan et al., 2021; Torres-Vega et al., 2020) |
| Boldine | 6.34 | 327.9 | 327.9 → 264.9 | 237.1 (57.1),  **264.9 (100),**  282.0 (49.8),  297.0 (82.9) | HMDB0039085^a^  MSBNK-RIKEN_NPDepo-NGA00419^b^  MSBNK-RIKEN-PR310581^b^  MSBNK-RIKEN-PR301114^b^  MSBNK-RIKEN-PR304997^b^  MSBNK-RIKEN-PR301122^b^  MSBNK-IPB_Halle-PB001521^b^  MSBNK-RIKEN-PR301106^b^  MSBNK-RIKEN_NPDepo-NGA00420^b^  MSBNK-RIKEN_NPDepo-CB000103 ^b^  (Castro-Saavedra et al., 2016) |
| Reticuline | 6.99 | 330.0 | 330.0 → 192.1 | 175.1 (29.5),  177.1 (30.0),  178.1 (7.1),  **192.1 (100)** | HMDB0003601^a^  MSBNK-mFam-MC12_000017^b^  MSBNK-mFam-MC12_000018^b^  PM010805^d^  MoNA016214^d^  MoNA016211^d^  CCMSLIB00005720178^d^  CCMSLIB00005720177^d^  CCMSLIB00005436048^d^  (Castro-Saavedra et al., 2016) |
| Isocorydine | 8.26 | 342.0 | 342.0 → 278.9 | 165.0 (45.7),  189.1 (29.0),  **278.9 (100),**  311.0 (71.1) | HMDB0030184^a^  MSBNK-NaToxAq-NA002538^b^  MSBNK-NaToxAq-NA002539^b^  MSBNK-NaToxAq-NA002540^b^  MSBNK-NaToxAq-NA002929^b^  MSBNK-NaToxAq-NA002930^b^  MSBNK-NaToxAq-NA002931^b^  MSBNK-NaToxAq-NA002932^b^  (Castro-Saavedra et al., 2016) |
| Laurotetanine  (Litsoeine) | 10.09 | 328.0 | 328.0 → 310.9 | 205.1 (79.7),  264.9 (44.6),  280.0 (55.7),  **310.9 (100)** | HMDB0030220^a^  Reference7553^c^  CCMSLIB00005436056^d^  (Castro-Saavedra et al., 2016; Nikolić et al., 2012; Torres-Vega et al., 2020) |
| *N*-Methyl-laurotetanine  (Lauroscholtzine or  Rogersine) | 10.46 | 342.0 | 342.0 → 311.1 | 237.1 (45.2),  265.0 (45.0),  280.0 (75.5),  311.1 (100) | CCMSLIB00005436059^d^  (Castro-Saavedra et al., 2016; Torres-Vega et al., 2020; Fedurco et al., 2015) |

RT = Retention time; rel. int. = relative intensity; Ref. = references; ^a^hmdb.ca; ^b^MassBank.eu; ^c^mzCloud.org; ^d^mona.fiehnlab.ucdavis.edu.

**Table 2S.** ^1^H-NMR data of aporphine alkaloids studied.

| **Hydrogen** | **^1^H-NMR chemical shift (ppm)** | | | | | |
| --- | --- | --- | --- | --- | --- | --- |
|  | **BOL^1^** | **LTS^1^** | **LTT^1^** | **NML^1^** | **DAB^2^** | **3Br-BOL^3^** |
| H-3 | 6.64 (s) | 6.63 (s) | 6.59 (s) | 6.55 (s) | 6.92 (s) | - |
| H-8 | 6.83 (s) | 6.78 (s) | 6.76 (s) | 6.73 (s) | 7.11 (s) | 6.75* (s) |
| H-11 | 7.89 (s) | 7.91 (s) | 8.07 (s) | 8.02 (s) | 7.92 (s) | 7.81 (s) |
| CH_3_O-1 | 3.60 (s) | 3.57 (s) | 3.66 (s) | 3.64 (s) | 3.58 (s) | 3.50 (s) |
| CH_3_O-2 | - | - | 3.88 (s) | 3.82 (s) | - | - |
| CH_3_O-9 | - | - | - | - | - | - |
| CH_3_O-10 | 3.91 (s) | 3.77 (s) | 3.88 (s) | 3.84 (s) | 3.79 (s) | 3.78 (s) |
| N-CH_3_ | 2.53 (s) | - | - | 2.51 (s) | 2.55 (s) | 2.39 (s) |
| OCOCH_3_-2 | - | - | - | - | 2.34 (s)* | - |
| OCOCH_3_-9 | - | - | - | - | 2.36 (s)* | - |

*Interchangeable assignments, ^1^(Guinaudeau et al., 1975), ^2^(Hara et al., 1995), and ^3^(Sobarzo-Sánchez et al., 2000).

**Table 3S.** ^1^H-NMR data of tetrahydroisoquinoline alkaloids studied.

| **Hydrogen** | **Chemical shift (ppm)** | | |
| --- | --- | --- | --- |
|  | **CC^1^** | **NMCC^2^** | **RET^3^** |
| H-5 | 6.86 (s) | 6.87 (s) | 6.53 (s) |
| H-8 | 6.49 (s) | 6.50 (s) | 6.34 (s) |
| H-5´ | 6.84 (dd, *J* = 8.3, 1.9 Hz | 6.85 (dd, *J* = 8.2, 1.8 Hz) | 7.72 (dd, *J* = 8.2, 1.8 Hz) |
| H-2´ | 6.53 (d, *J* = 1.9 Hz) | 6.54 (d, *J* = 1.9 Hz) | 6.74 (d, *J* = 1.9 Hz) |
| H-6´ | 6.57 (d, *J* = 8.2 Hz) | 6.56 (d, *J* = 8.2 Hz) | 6.58 (d, *J* = 8.2 Hz) |
| CH_3_O-6 | 3.80 (s) | 3.78 (s) | 3.83* (s) |
| CH_3_O-4´ | 3.80 (s) | 3.78 (s) | 3.83* (s) |
| N-CH_3_ | - | 2.43 (s) | 2.46 (s) |

*Interchangeable assignments,^1^(Al-ghazzawi, 2019), ^2^(Tomita, M. Kugo, 1956),  ^3^(Castro-Saavedra et al., 2016).

**Table 4S.** PAINS and Brenks alerts obtained from the SwissADME platform.

| **Inhibitor** | **Acronym** | **PAINS** | **Brenks** |
| --- | --- | --- | --- |
| (*R*,*S*)-Coclaurine | CC | 0 Alerts | 0 Alerts |
| (*R*,*S*)-*N*-methylcoclaurine | NMCC | 0 Alerts | 0 Alerts |
| Reticuline | RET | 0 Alerts | 0 Alerts |
| Laurolitsine | LTS | 0 Alerts | 0 Alerts |
| Boldine | BOL | 0 Alerts | 0 Alerts |
| Laurotetanine | LTT | 0 Alerts | 0 Alerts |
| *N*-methyllaurotetanine | NML | 0 Alerts | 0 Alerts |
| Isocorydine | ISO | 0 Alerts | 0 Alerts |
| Diacetylboldine | DAB | 0 Alerts | 1 Alert Phenol ester |
| 3-Bromoboldine | 3BrBOL | 0 Alerts | 0 Alerts |
| Gallic acid | GA | 1 Alert catechol | 1 Alert catechol |
| Arbutin | ARB | 0 Alerts | 0 Alerts |
| Kojic acid | KA | 0 Alerts | 0 Alerts |

**Table 5S.** Pharmacokinetic properties of natural and hemisynthetic alkaloids from *Peumus boldus, Cryptocarya alba,* and *Laurelia sempervirens*.

| **Property** | **CC** | **NMCC** | **RET** | **LTS** | **BOL** | **LTT** | **ISO** | **DAB** | **3BrBOL** | **GA** | **ARB** | **KA** |
| --- | --- | --- | --- | --- | --- | --- | --- | --- | --- | --- | --- | --- |
| **MF** | C_17_H_19_NO_3_ | C_18_H_21_NO_3_ | C_19_H_23_NO_4_ | C_18_H_19_NO_4_ | C_19_H_21_NO_4_ | C_20_H_23_NO_4_ | C_20_H_23_NO_4_ | C_23_H_25_NO_6_ | C_19_H_20_BrNO_4_ | C_7_H_6_O_5_ | C_12_H_16_O_7_ | C_6_H_6_O_4_ |
| **MW** | 285.34 | 299.36 | 329.39 | 313.35 | 327.37 | 341.4 | 341.4 | 411.05 | 406.27 | 170.12 | 272.25 | 142.11 |
| **ROT** | 3 | 3 | 4 | 2 | 2 | 3 | 3 | 6 | 2 | 1 | 3 | 1 |
| **HBA** | 4 | 4 | 5 | 5 | 5 | 5 | 5 | 7 | 5 | 5 | 7 | 4 |
| **HBD** | 3 | 2 | 2 | 3 | 2 | 1 | 1 | 0 | 2 | 4 | 5 | 2 |
| **MR** | 85.62 | 90.52 | 97.01 | 91.1 | 96 | 100.47 | 100.47 | 114.96 | 103.7 | 39.47 | 62.61 | 33.13 |
| **TPSA** | 61.72 | 52.93 | 62.16 | 70.95 | 62.16 | 51.16 | 51.16 | 74.30 | 62.16 | 97.99 | 119.61 | 70.67 |
| **LogP** | 2.55 | 2.78 | 3.13 | 2.63 | 2.94 | 3.41 | 3.34 | 3.67 | 3.19 | 0.21 | 1.64 | 1.12 |
| **LogS** | -3.46 | -3.82 | -3.88 | -3.45 | -3.82 | -4.04 | -3.73 | -4.14 | -4.73 | -1.64 | -0.71 | -0.7 |
| **Class** | Soluble | Soluble | Soluble | Soluble | Soluble | Moderately soluble | Soluble | Moderately soluble | Moderately soluble | Very soluble | Very soluble | Very soluble |
| **GI** | High | High | High | High | High | High | High | High | High | High | High | High |
| **BBB** | Yes | Yes | Yes | Yes | Yes | Yes | Yes | Yes | Yes | No | No | No |
| **P-gp** | Yes | Yes | Yes | Yes | Yes | Yes | Yes | No | Yes | No | No | No |
| **CYP_1A2_** | No | No | No | Yes | Yes | Yes | Yes | No | Yes | No | No | No |
| **CYP_2C19_** | No | No | No | No | No | No | No | Yes | No | No | No | No |
| **CYP_2C9_** | No | No | No | No | No | No | No | Yes | No | No | No | No |
| **CYP_2D6_** | Yes | Yes | Yes | Yes | Yes | Yes | Yes | Yes | Yes | No | No | No |
| **CYP_3A4_** | No | No | No | Yes | Yes | Yes | Yes | Yes | Yes | Yes | No | No |
| **LogKp** | -6.21 | -5.97 | -6.17 | -6.61 | -6.37 | -6.22 | -6.56 | -6.73 | -6.36 | -6.84 | -8.92 | -7.62 |
| **Lipinski** | 0 | 0 | 0 | 0 | 0 | 0 | 0 | 0 | 0 | 0 | 0 | 0 |
| **BS** | 0.55 | 0.55 | 0.55 | 0.55 | 0.55 | 0.55 | 0.55 | 0.55 | 0.55 | 0.56 | 0.55 | 0.55 |

CC: (*R,S*)-Coclaurine; NMCC: (*R,S*)-*N*-Methylcoclaurine; RET: Reticuline; LTS: Laurolitsine; BOL: Boldine; LTT: Laurotetanine; ISO: Isocorydine; DAB: Diacetylboldine; 3BrBOL: 3-Bromoboldine, GA: Gallic acid, ARB: Arbutin; KA: Kojic acid; MF: Molecular formula; MW: Molecular weight (g/mol); Rot: Rotatable bonds; HBA: H-bond acceptors; HBD: H-bond donors; MR: Molar Refractivity; TPSA: Topological Polar Surface Area (Å²); LogP o/w: Logarithmic function of Partition coefficient oil/water (iLOGP); LogS: Logarithmic function of water solubility (ESOL); Class: Classification of water solubility. GI: Gastrointestinal absorption; BBB: Blood Brain Barrier permeant; P-gp: P-glycoprotein substrate; CYP1A2: Cytochrome 1A2 inhibitor; CYP2C19: Cytochrome 2C19 inhibitor; CYP2C9: Cytochrome 2C9 inhibitor; CYP2D6: Cytochrome 2D6 inhibitor; CYP3A4: Cytochrome 3A4 inhibitor; LogKp: skin permeation, cm/s; Lipinski: Violation of Lipinski’s Rules: BS: Bioavailability Score.


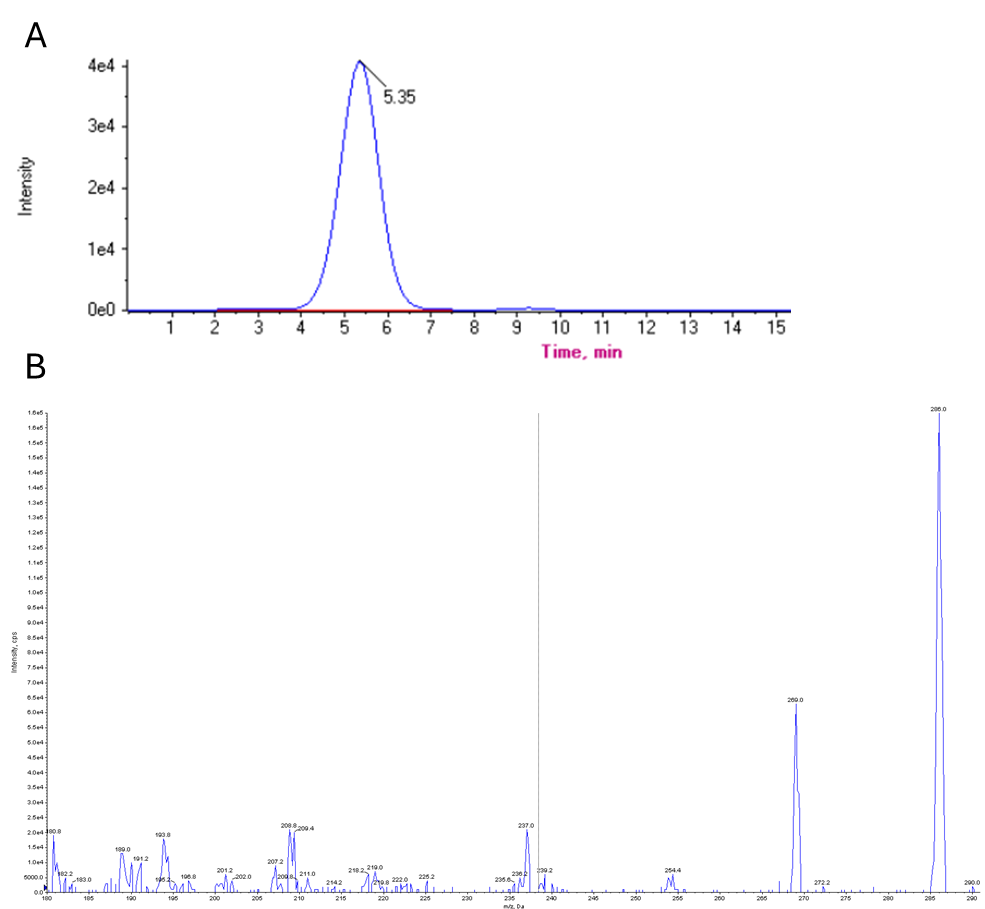


**Figure 1S.** (A) UHPLC chromatogram of coclaurine (RT = 5.35 min). (B) MS/MS spectrum of coclaurine.


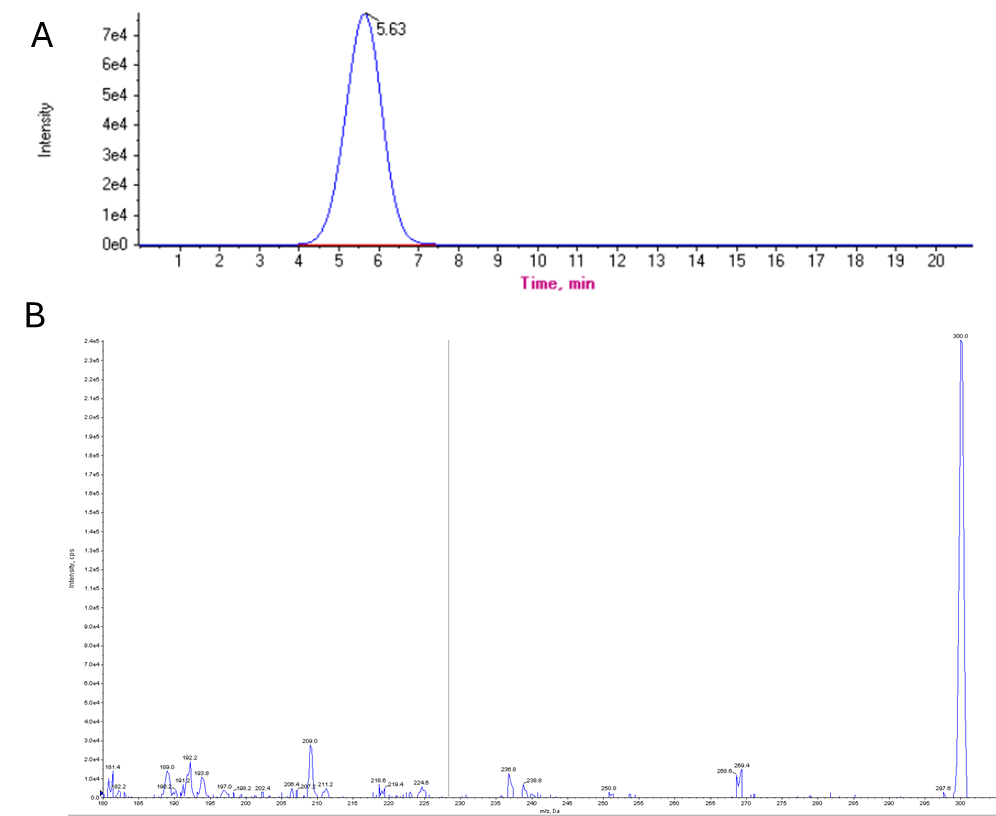


**Figure 2S.** (A) UHPLC chromatogram of *N*-methylcoclaurine (RT = 5.63 min). (B) MS/MS spectrum of *N*-methylcoclaurine.


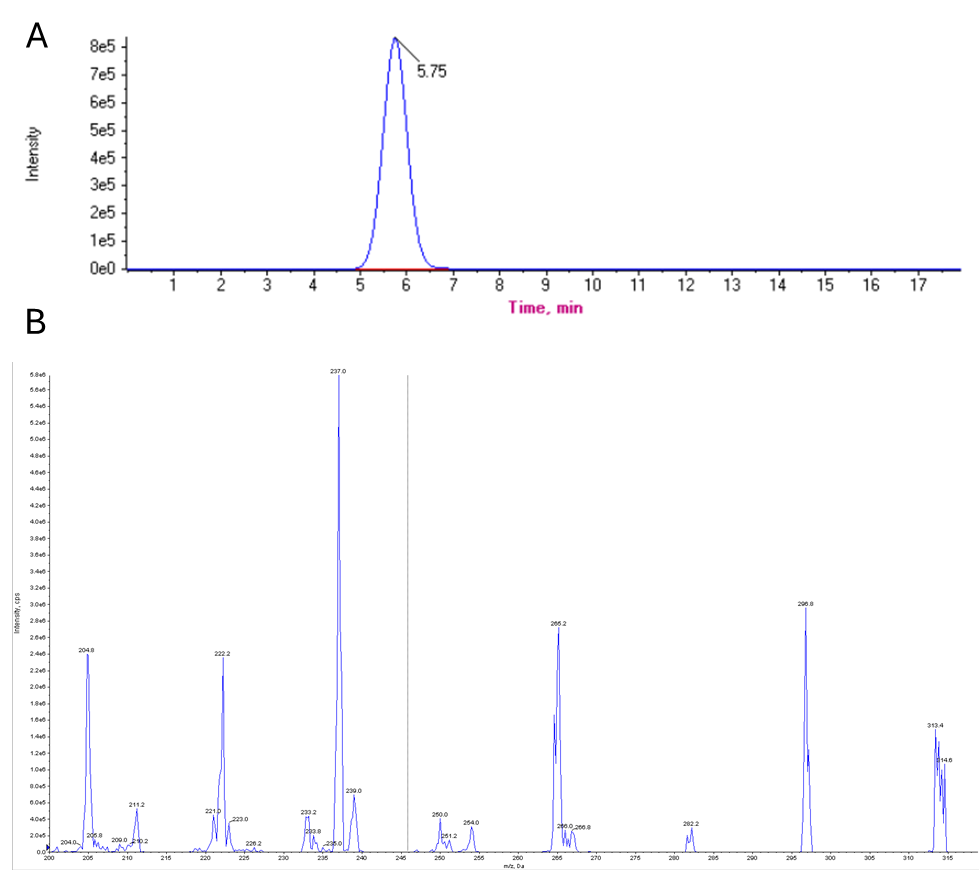


**Figure 3S.** (A) UHPLC chromatogram of laurolitsine (RT = 5.75 min). (B) MS/MS spectrum of laurolitsine.


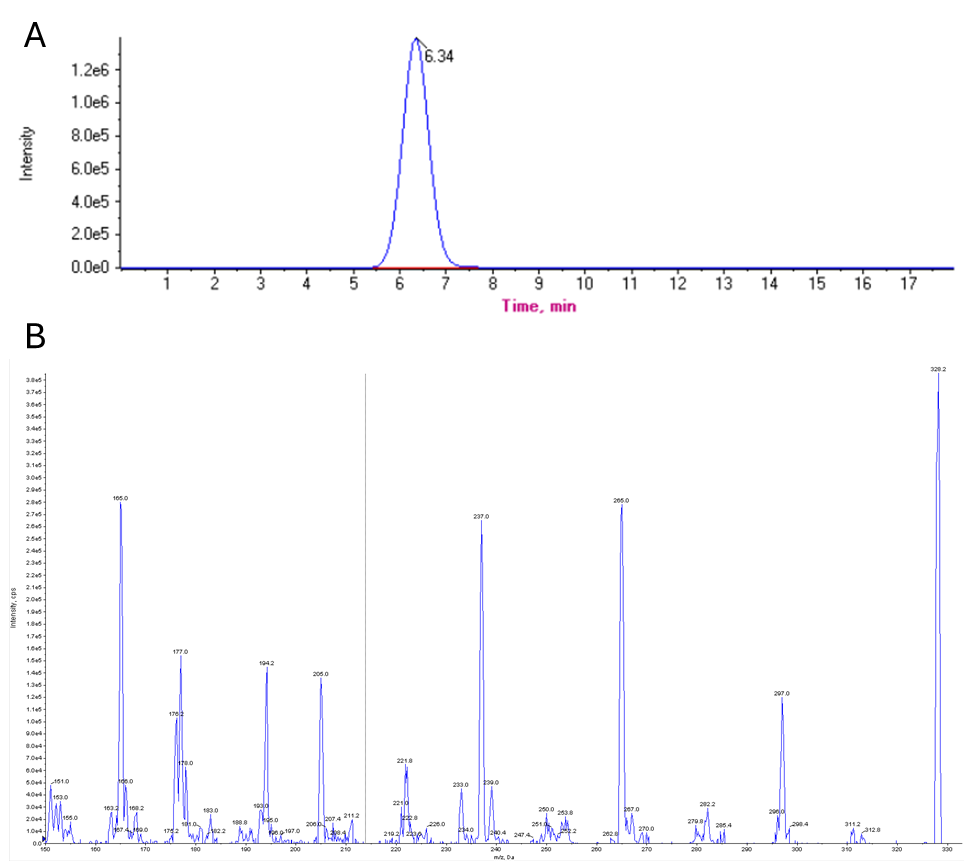


**Figure 4S.** (A) UHPLC chromatogram of boldine (RT = 6.34 min). (B) MS/MS spectrum of boldine.


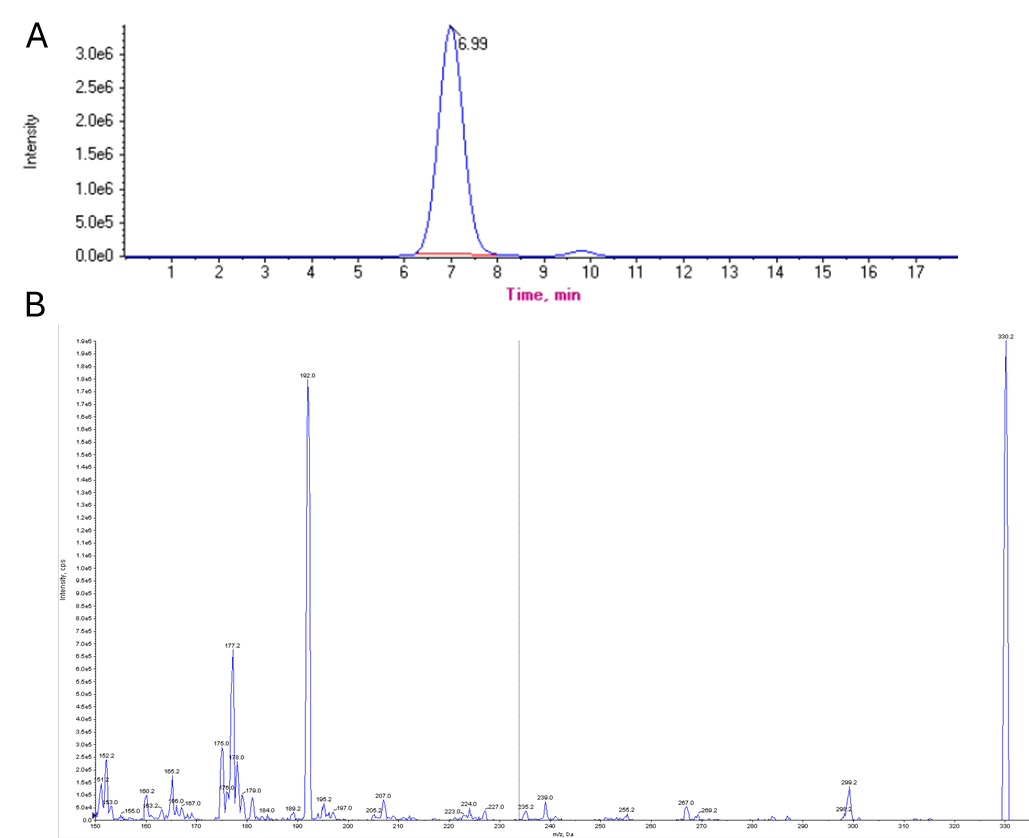


**Figure 5S.** (A) UHPLC chromatogram of reticuline (RT = 6.99 min). (B) MS/MS spectrum of reticuline.


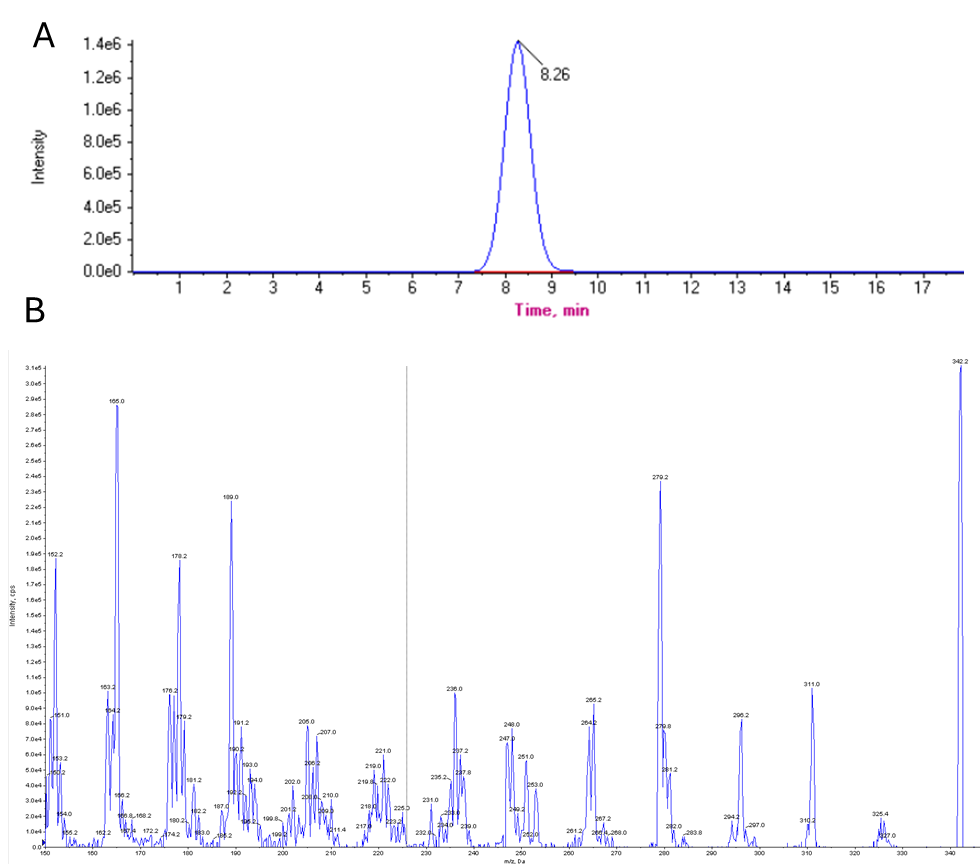


**Figure 6S.** (A) UHPLC chromatogram of isocorydine (RT = 10.46 min). (B) MS/MS spectrum of isocorydine.


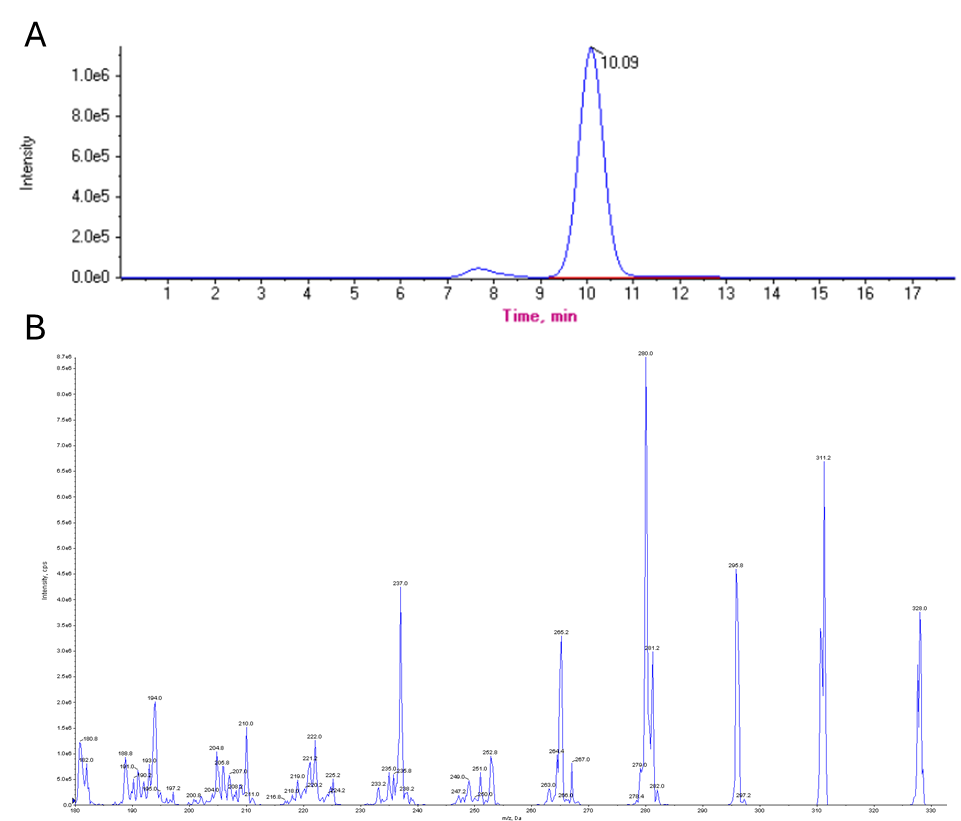


**Figure 7S.** (A) UHPLC chromatogram of laurotetanine (RT = 10.09 min). (B) MS/MS spectrum of laurotetanine.


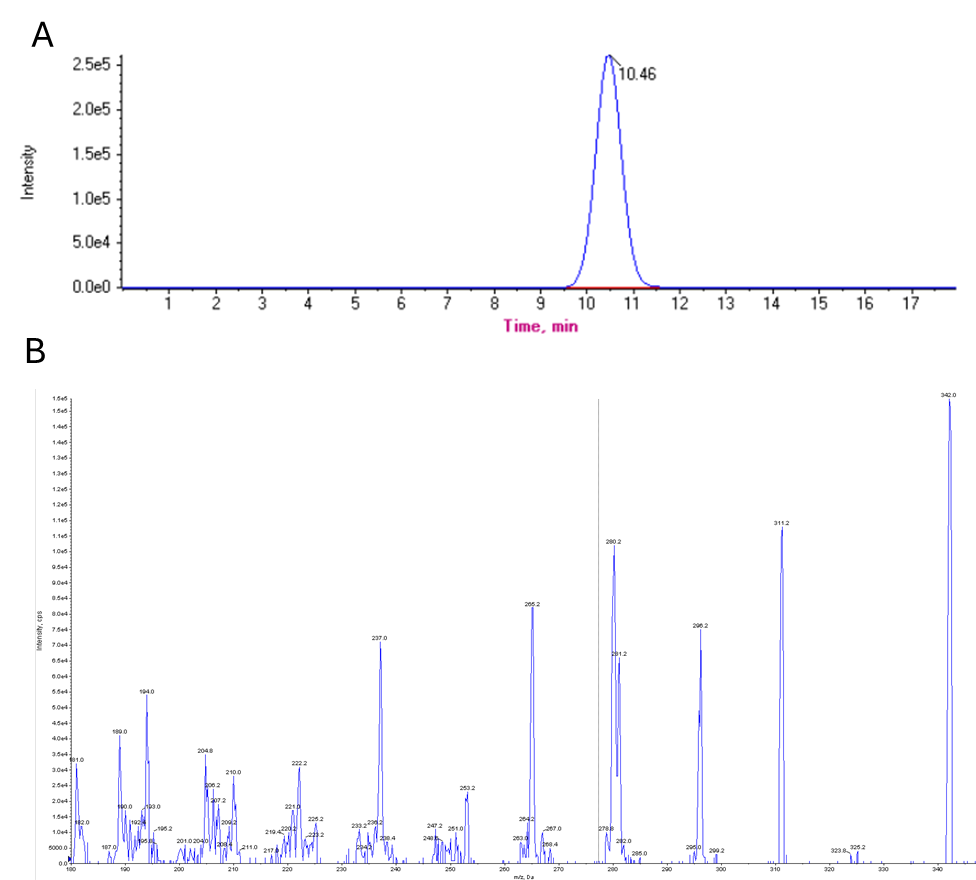


**Figure 8S.** (A) UHPLC chromatogram of *N*-methyl-laurotetanine (RT = 10.46 min). (B) MS/MS spectrum of *N*-methyl-laurotetanine.

**Figure 9S.** Melanin standard curve for melanin determination in cell lysates.


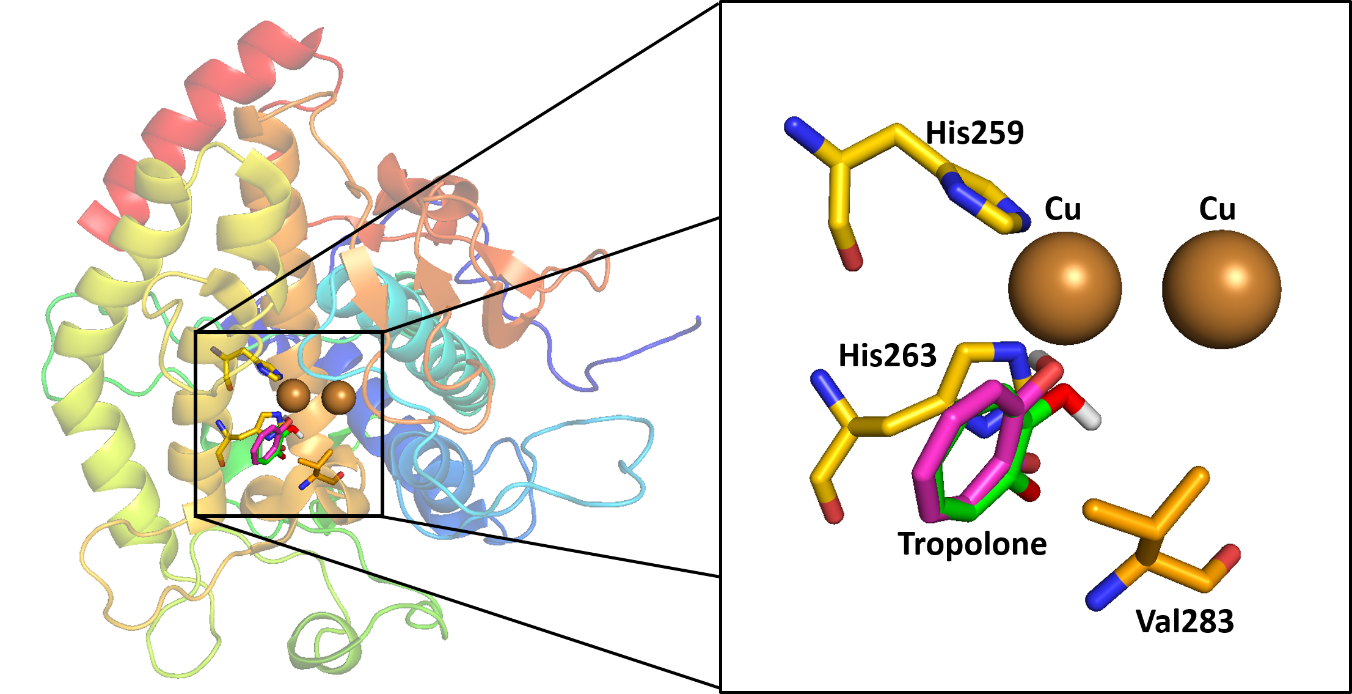


**Figure 10S.** Visualization of molecular docking results for the crystalized ligand tropolone and scale-up within the active site of the tyrosinase enzyme (PDB ID: 2Y9X). Color code: Magenta: carbon atoms for tropolone structure docked. Green: carbon atoms for tropolone structure docked. Yellow: carbon atoms of the amino acids being to the active site. Red: oxygen atoms. Blue: nitrogen atoms. White: polar hydrogen atom.


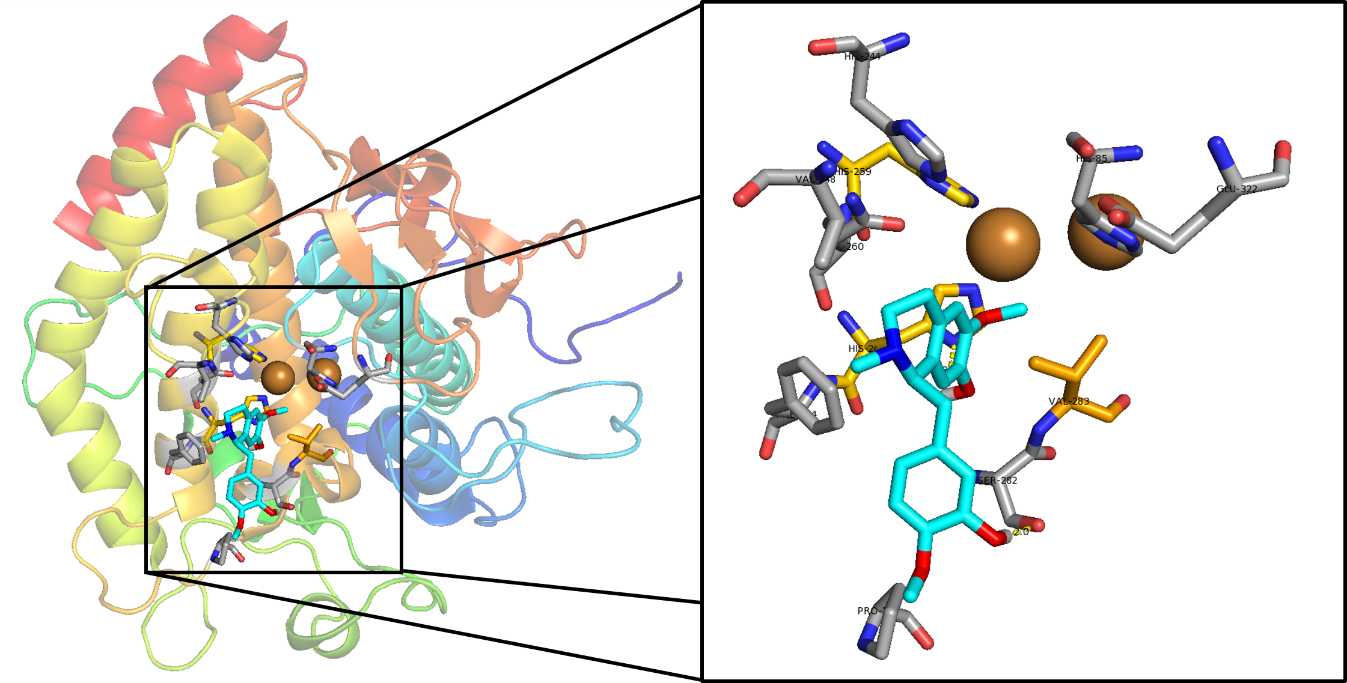


**Figure 11S.** Visualization of molecular docking results for the alkaloid reticuline and scale-up within the active site of the tyrosinase enzyme (PDB ID: 2Y9X). Color code: Cyan: carbon atoms for the docked alkaloid. Yellow: carbon atoms of the amino acids being to the active site. Grey: carbon atoms that are close to the active site and participate in the stabilization of the natural alkaloid. Red: oxygen atoms. Blue: nitrogen atoms. White: polar hydrogen atom. Dashed yellow line: hydrogen bonding between ligand and amino acid from the active site.


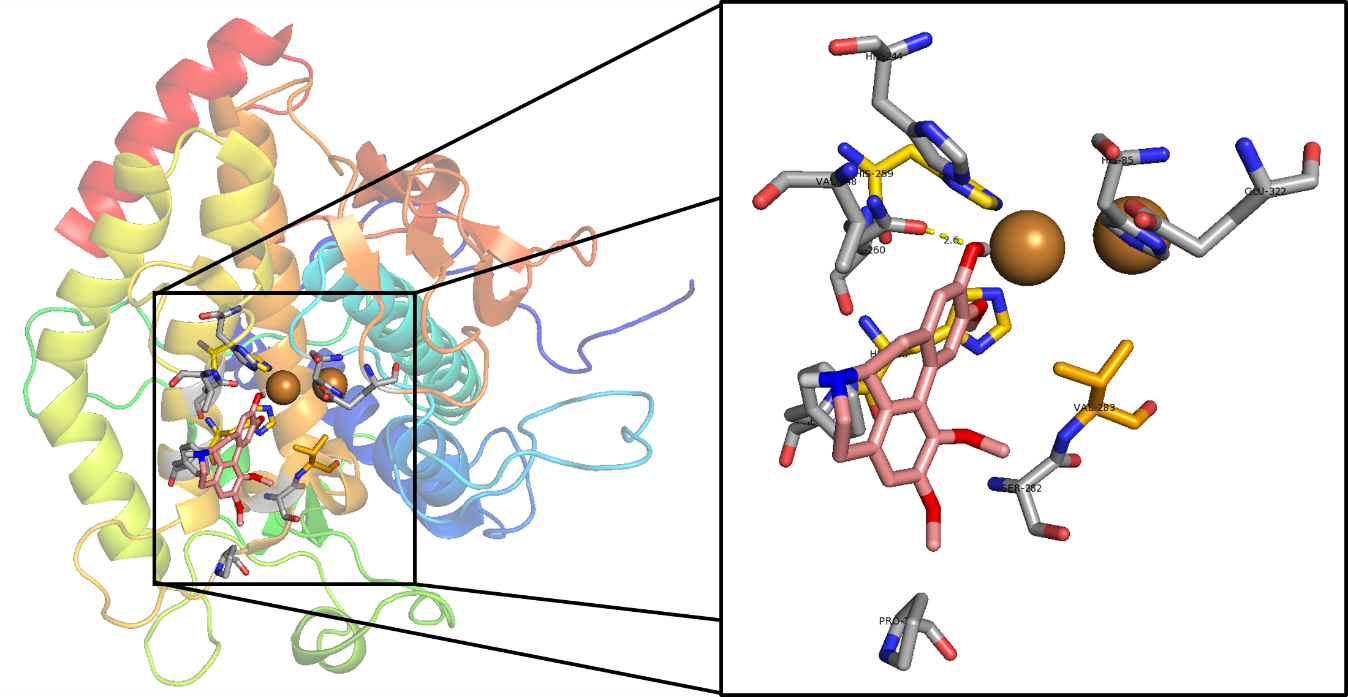


**Figure 12S.** Visualization of molecular docking results for the alkaloid laurotetanine and scale-up within the active site of the tyrosinase enzyme (PDB ID: 2Y9X). Color code: Salmon: carbon atoms for the docked alkaloid. Yellow: carbon atoms of the amino acids being to the active site. Grey: carbon atoms that are close to the active site and participate in the stabilization of the natural alkaloid. Red: oxygen atoms. Blue: nitrogen atoms. White: polar hydrogen atom. Dashed yellow line: hydrogen bonding between ligand and amino acid from the active site.


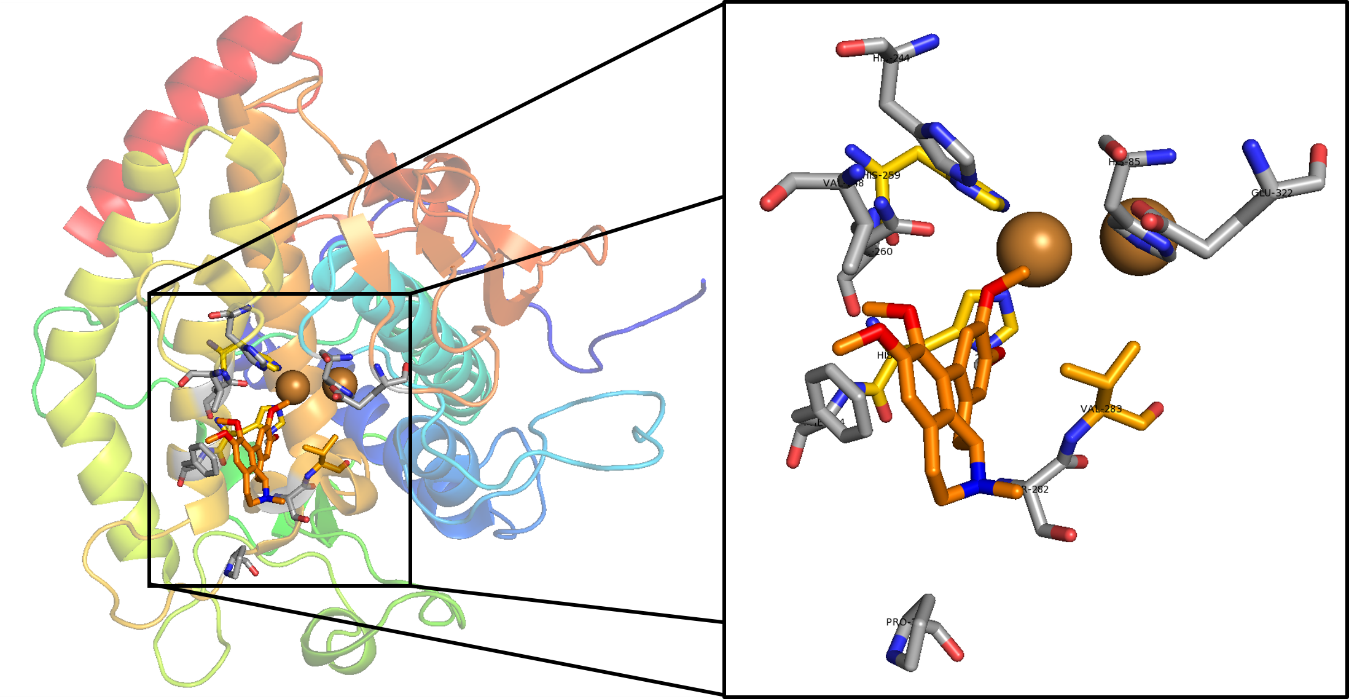


**Figure 13S.** Visualization of molecular docking results for the alkaloid *N*-methyllaurotetanine and scale-up within the active site of the tyrosinase enzyme (PDB ID: 2Y9X). Color code: Orange: carbon atoms for the docked alkaloid. Yellow: carbon atoms of the amino acids being to the active site. Grey: carbon atoms that are close to the active site and participate in the stabilization of the natural alkaloid. Red: oxygen atoms. Blue: nitrogen atoms. White: polar hydrogen atom. Dashed yellow line: hydrogen bonding between ligand and amino acid from the active site.


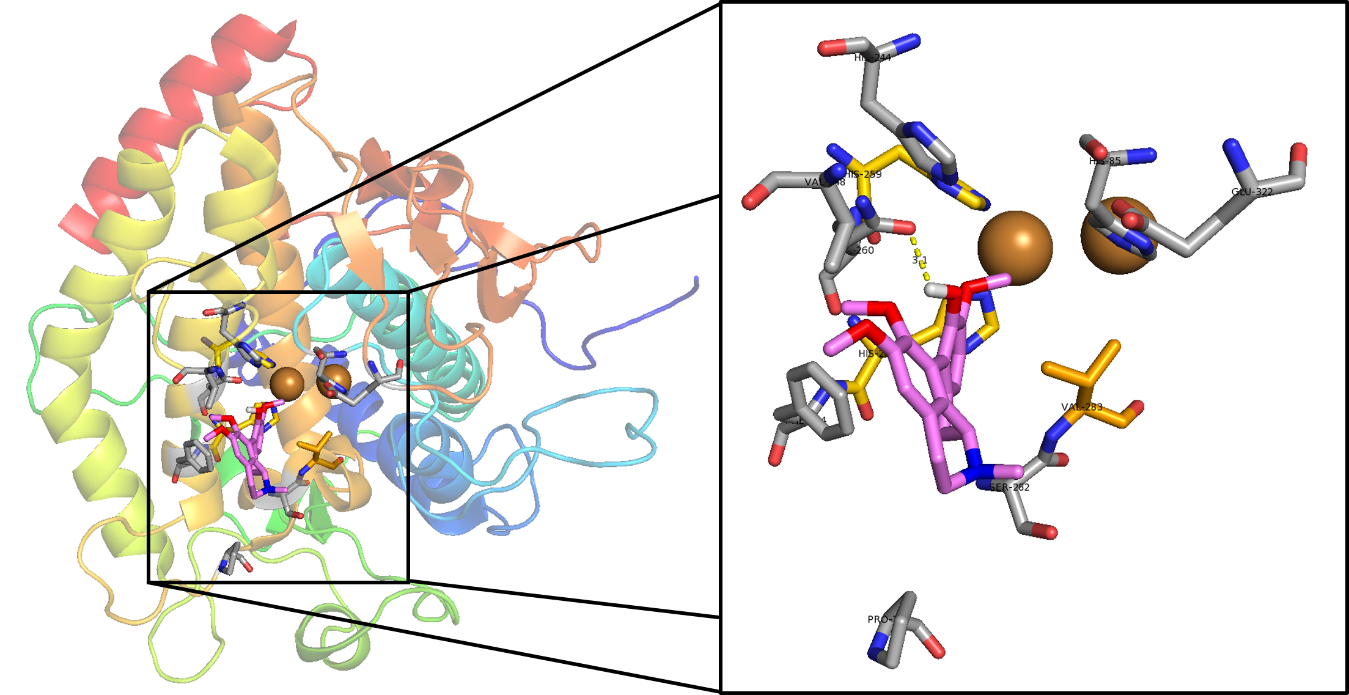


**Figure 14S.** Visualization of molecular docking results for the alkaloid isocorydine and scale-up within the active site of the tyrosinase enzyme (PDB ID: 2Y9X). Color code: Violet: carbon atoms for the docked alkaloid. Yellow: carbon atoms of the amino acids being to the active site. Grey: carbon atoms that are close to the active site and participate in the stabilization of the natural alkaloid. Red: oxygen atoms. Blue: nitrogen atoms. White: polar hydrogen atom. Dashed yellow line: hydrogen bonding between ligand and amino acid from the active site.


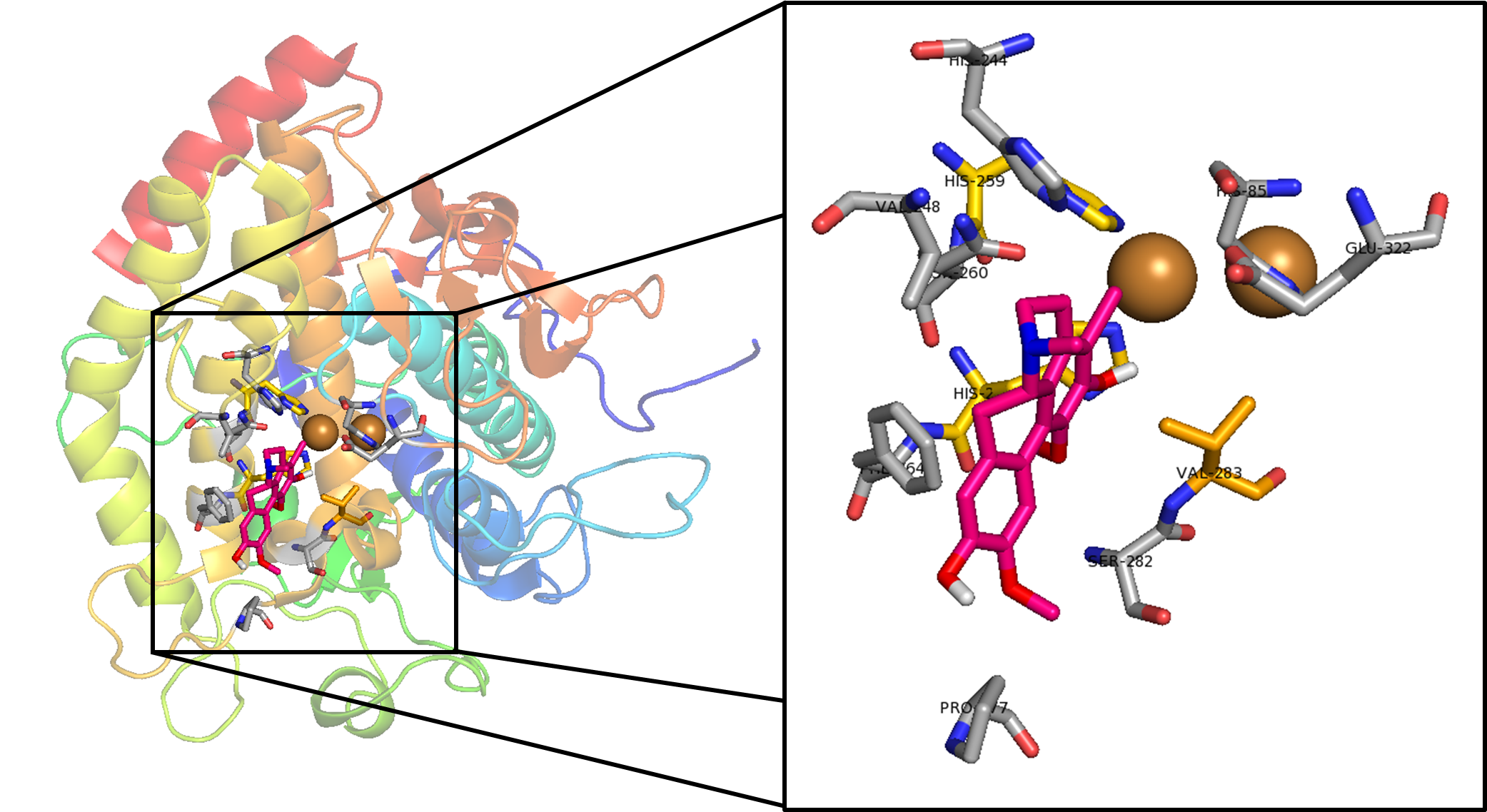


**Figure 15S.** Visualization of molecular docking results for the alkaloid 3-bromoboldine and scale-up within the active site of the tyrosinase enzyme (PDB ID: 2Y9X). Color code: Hotpink: carbon atoms for the docked alkaloid. Yellow: carbon atoms of the amino acids being to the active site. Grey: carbon atoms that are close to the active site and participate in the stabilization of the natural alkaloid. Red: oxygen atoms. Blue: nitrogen atoms. White: polar hydrogen atom. Dashed yellow line: hydrogen bonding between ligand and amino acid from the active site.


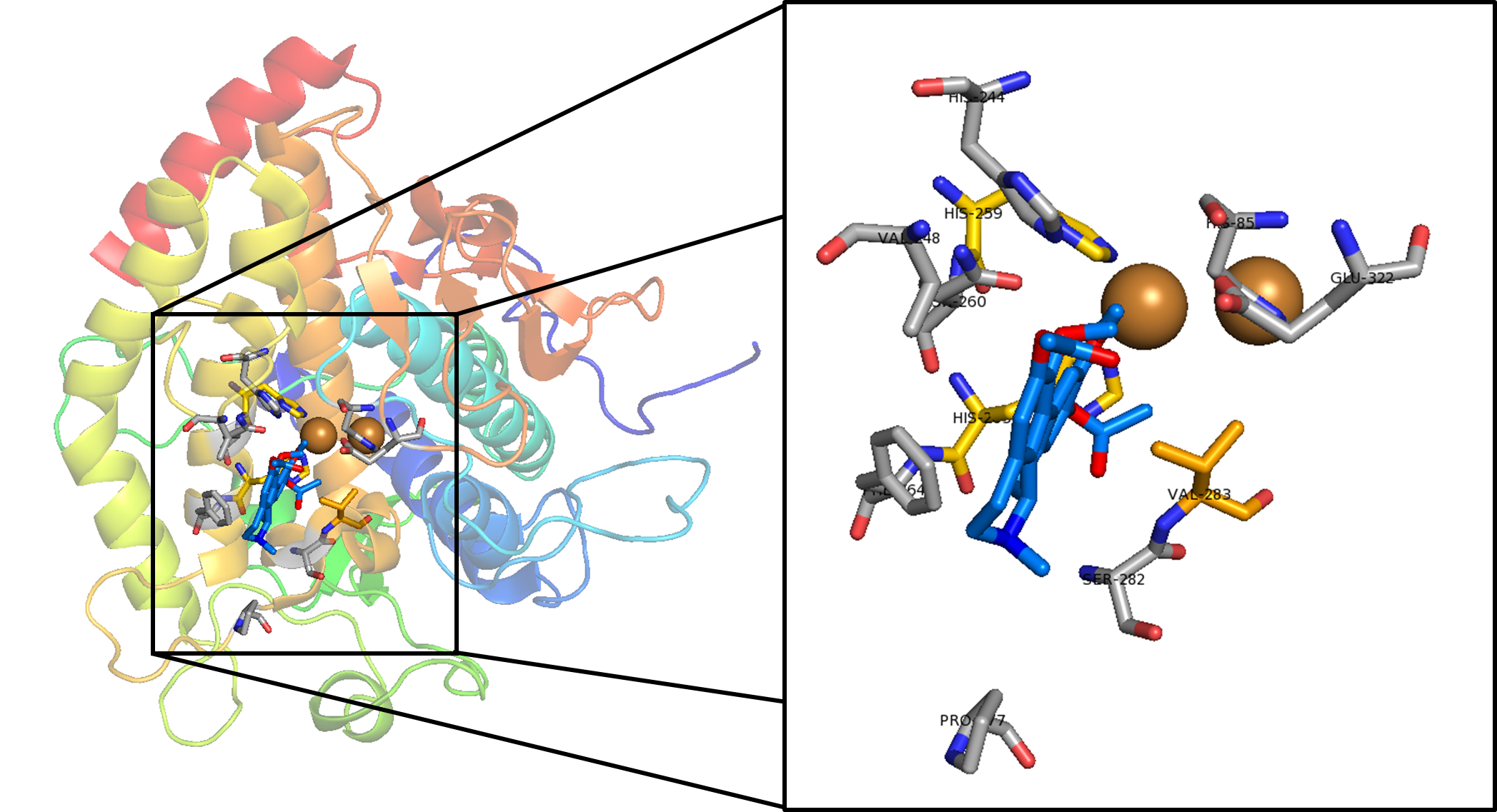


**Figure 16S.** Visualization of molecular docking results for the alkaloid diacetylboldine and scale-up within the active site of the tyrosinase enzyme (PDB ID: 2Y9X). Color code: Marine: carbon atoms for the docked alkaloid. Yellow: carbon atoms of the amino acids being to the active site. Grey: carbon atoms that are close to the active site and participate in the stabilization of the natural alkaloid. Red: oxygen atoms. Blue: nitrogen atoms. White: polar hydrogen atom. Dashed yellow line: hydrogen bonding between ligand and amino acid from the active site.

**References**

Al-ghazzawi, A. M. (2019). Anti-cancer activity of new benzyl isoquinoline alkaloid from Saudi plant Annona squamosa. *BMC Chem.* 13, 13. doi: 10.1186/s13065-019-0536-4

Castro-Saavedra, S., Fuentes-Barros, G., Tirapegui, C., Acevedo-Fuentes, Wi., Cassels, B. K., Barriga, A., et al. (2016). Phytochemical analysis of alkaloids from the Chilean endemic tree Cryptocarya alba. *J. Chil. Chem. Soc.* 61, 3076–3080. doi: 10.4067/S0717-97072016000300014

Fedurco, M., Gregorová, J., Šebrlová, K., Kantorová, J., Peš, O., Baur, R., et al. (2015). Modulatory Effects of Eschscholzia californica Alkaloids on Recombinant GABA A Receptors. *Biochem. Res. Int.* 2015, 1–9. doi: 10.1155/2015/617620

Guinaudeau, H., Leboeuf, M., and Cave, A. (1975). Aporphine alkaloids. *Lloydia* 38, 275–338. Available at: http://www.ncbi.nlm.nih.gov/pubmed/241890

Hara, H., Kaneko, K., Endoh, M., Uchida, H., and Hoshino, O. (1995). A novel ring cleavage and recyclization of N-cyanomethyl-1,2,3,4-tetrahydroisoquinolinium methiodides: A biomimetic synthesis of litebamine. *Tetrahedron* 51, 10189–10204. doi: 10.1016/0040-4020(95)00614-E

Nikolić, D., Gödecke, T., Chen, S.-N., White, J., Lankin, D. C., Pauli, G. F., et al. (2012). Mass spectrometric dereplication of nitrogen-containing constituents of black cohosh (Cimicifuga racemosa L.). *Fitoterapia* 83, 441–460. doi: 10.1016/j.fitote.2011.12.006

Schmidt, J., Raith, K., Boettcher, C., and Zenk, M. H. (2005). Analysis of Benzylisoquinoline-Type Alkaloids by Electrospray Tandem Mass Spectrometry and Atmospheric Pressure Photoionization. *Eur. J. Mass Spectrom.* 11, 325–333. doi: 10.1255/ejms.745

Sobarzo-Sánchez, E. M., Arbaoui, J., Protais, P., and Cassels, B. K. (2000). Halogenated Boldine Derivatives with Enhanced Monoamine Receptor Selectivity. *J. Nat. Prod.* 63, 480–484. doi: 10.1021/np990433j

Tan, Y.-F., Wang, R.-Q., Wang, W.-T., Wu, Y., Ma, N., Lu, W.-Y., et al. (2021). Study on the pharmacokinetics, tissue distribution and excretion of laurolitsine from Litsea glutinosa in Sprague-Dawley rats. *Pharm. Biol.* 59, 882–890. doi: 10.1080/13880209.2021.1944221

Tian, W., Zhi, H., Yang, C., Wang, L., Long, J., Xiao, L., et al. (2018). Chemical composition of alkaloids of Plumula nelumbinis and their antioxidant activity from different habitats in China. *Ind. Crops Prod.* 125, 537–548. doi: 10.1016/j.indcrop.2018.09.045

Tomita, M. Kugo, T. (1956). Alkaloids of Berberidaceous plants - XIX: Alkaloids of B. tschonoskyana I. Isolation of bases. *Yakugak Zasshi* 79, 317–321.

Torres-Vega, J., Gómez-Alonso, S., Pérez-Navarro, J., and Pastene-Navarrete, E. (2020). Green Extraction of Alkaloids and Polyphenols from Peumus boldus Leaves with Natural Deep Eutectic Solvents and Profiling by HPLC-PDA-IT-MS/MS and HPLC-QTOF-MS/MS. *Plants* 9, 242. doi: 10.3390/plants9020242
